# A highly-sensitive genetically encoded temperature indicator exploiting a temperature-responsive elastin-like polypeptide

**DOI:** 10.1038/s41598-021-96049-5

**Published:** 2021-08-13

**Authors:** Cong Quang Vu, Shun-ichi Fukushima, Tetsuichi Wazawa, Takeharu Nagai

**Affiliations:** 1 Graduate School of Frontier Biosciences, Osaka University, Suita, Osaka 565-0871 Japan; 2 SANKEN (The Institute of Scientific and Industrial Research), Osaka University, Ibaraki, Osaka 567-0047 Japan

**Keywords:** Biological techniques, Biophysics, Cell biology, Chemical biology

## Abstract

Genetically encoded temperature indicators (GETIs) allow for real-time measurement of subcellular temperature dynamics in live cells. However, GETIs have suffered from poor temperature sensitivity, which may not be sufficient to resolve small heat production from a biological process. Here, we develop a highly-sensitive GETI, denoted as ELP-TEMP, comprised of a temperature-responsive elastin-like polypeptide (ELP) fused with a cyan fluorescent protein (FP), mTurquoise2 (mT), and a yellow FP, mVenus (mV), as the donor and acceptor, respectively, of Förster resonance energy transfer (FRET). At elevated temperatures, the ELP moiety in ELP-TEMP undergoes a phase transition leading to an increase in the FRET efficiency. In HeLa cells, ELP-TEMP responded to the temperature from 33 to 40 °C with a maximum temperature sensitivity of 45.1 ± 8.1%/°C, which was the highest ever temperature sensitivity among hitherto-developed fluorescent nanothermometers. Although ELP-TEMP showed sensitivity not only to temperature but also to macromolecular crowding and self-concentration, we were able to correct the output of ELP-TEMP to achieve accurate temperature measurements at a subcellular resolution. We successfully applied ELP-TEMP to accurately measure temperature changes in cells induced by a local heat spot, even if the temperature difference was as small as < 1 °C, and to visualize heat production from stimulated Ca^2+^ influx in live HeLa cells induced by a chemical stimulation. Furthermore, we investigated temperatures in the nucleus and cytoplasm of live HeLa cells and found that their temperatures were almost the same within the temperature resolution of our measurement. Our study would contribute to better understanding of cellular temperature dynamics, and ELP-TEMP would be a useful GETI for the investigation of cell thermobiology.

## Introduction

Fluorescence nanothermometry (FNT) enables us to monitor temperature dynamics at a high spatial and temporal resolution inside live cells. FNT uses a temperature-sensitive fluorescent indicator to measure the temperature through the fluorescence. Applications of FNT have led not only to exciting reports such as temperature difference between the nucleus and cytoplasm^1–3^, hot mitochondria^4^, and the low thermal conductivity in cells^5,6^ but also to controversy about a discrepancy between a theoretical calculation and experimental measurements of intracellular temperature changes in single cells^7–9^. There have been efforts in developing fluorescent temperature indicators for FNT using different temperature-responsive compounds including chemical dyes^10^, nanoparticles^11^, nanodiamonds^12^, polymers^1^, oligonucleotides^13^, and fluorescent proteins (FPs)^14^. For live-cell imaging, FP-based temperature indicators are particularly advantageous, because they can be genetically encoded so that they are targeted to a specific protein or can be localized to an organelle by fusing with a targeting peptide or protein. However, genetically encoded temperature indicators (GETIs) have often suffered from poor temperature sensitivity that may not be sufficient to resolve small heat production from biological processes^15^. Thus, a new GETI with high sensitivity to temperature is of great demand.

Elastin-like polypeptides (ELPs) are polypeptides that consist of a primary sequence of (VPGXG)_*n*_, where X is a guest amino acid residue except proline^16^ and *n* is the number of pentapeptide repeats^17^. One of their unique characteristics is the thermal responsiveness displaying the lower critical solution temperature (LCST) in an aqueous solution: they are in the soluble state below the transition temperature (*T*_t_) whereas they undergo a self-assembly process to form coacervate at an elevated temperature above *T*_t_^16,17^. The *T*_t_ of ELPs can be manipulated by changing the guest residue X and the number of the repeats *n*: *T*_t_ lowers as the guest residue X becomes more hydrophobic, and vice versa^18^; *T*_t_ lowers as the number of the repeats *n* becomes larger, and vice versa^17^. Additionally, ELPs can be genetically encoded and expressed in live cells. Thus, ELPs should be potential candidates as temperature-sensing domains for the development of GETIs. Previously, Mackay et al*.* utilized ELPs fused with a green FP (GFP) to detect intracellular temperature through ELP coacervation as observed by fluorescence microscopy^19^. However, this approach was able to detect only a temperature at which the ELP converted into the coacervate, and thus, did not provide the quantitative measurement of temperature. Chen et al*.* reported an ELP labeled with a hydrophobicity-sensitive fluorescent dye, which showed high fluorescence upon the coacervation of ELP caused by the elevation of temperature above its *T*_t_^20^. Because this approach needed fluorescence labeling of the ELP to produce the temperature indicator and its delivery into cells, the scope of the application was rather limited. To move a step forward from the ELP-derived fluorescent temperature indicators thus far, it is desirable to develop a novel GETI that takes the advantage of the highly temperature-sensitive behavior of ELPs to perform quantitative temperature measurement at a high accuracy, and is ready to be observed after expression in cells without any chemical modification.

Förster resonance energy transfer (FRET) has been extensively applied to the development of genetically encoded fluorescent indicators. A general design of genetically encoded FRET-based indicators involves two FPs of different excitation and emission wavelengths as the donor and acceptor of FRET, and a sensor domain that detects a factor of interest, e.g., ions, macromolecular crowding, or temperature. Upon excitation of the donor, the excitation energy is partly or entirely transferred from the donor to the acceptor to undergo fluorescence emission of the acceptor as well as that of the donor, depending on the FRET efficiency^21^. Because the FRET efficiency is dependent on parameters such as the distance and relative orientation between the donor and acceptor, when the conformation of the sensor domain changes upon the detection of a factor, the distance or the relative orientation changes so that the FRET efficiency alters, leading to changes of the fluorescence intensities of the donor and acceptor. It should be noted that the ratio of the fluorescence intensities between the donor and acceptor can be a measure of the FRET efficiency, and thus, FRET-based indicators are useful to perform ratiometric imaging with the donor and acceptor fluorescence. In fact, a number of genetically encoded indicators have applied FRET to sensing ions (Ca^2+^^22^, Mg^2+^^23^, K^+^^24^, and pH^25^), membrane potential^26^, and macromolecular crowding^27^. Thus, if donor and acceptor FPs are fused with an ELP, this fusion protein is most likely to show a large change of FRET efficiency in responding to temperature changes around its *T*_t_ to achieve a high sensitivity to temperature.

In this study, we developed the first FRET-based GETI from an ELP and a FRET pair of cyan FP, mTurquoise2 (mT)^28^, and yellow FP, mVenus (mV)^29^. The ELP moiety in the present GETI undergoes a phase transition around a physiological temperature of mammalian cells (33–40 °C), and this leads to a large change of FRET efficiency. Although the fluorescence signal of the present GETI was found to be affected by factors such as macromolecular crowding and self-concentration, we show that we are able to accurately measure temperature at a subcellular resolution by properly calibrating the GETI. We demonstrate that in HeLa cells expressing the GETI, we were able to detect a quick temperature rise on the sub-second time scale induced by transient heat supply from a local heat source and a temperature increase lasting for > 100 s induced by a drug stimulation. Furthermore, we investigated temperatures in the nucleus and cytoplasm of live HeLa cells.

## Results

### Development of ELP-based temperature indicators (ELP-TEMP)

**Figure 1:**
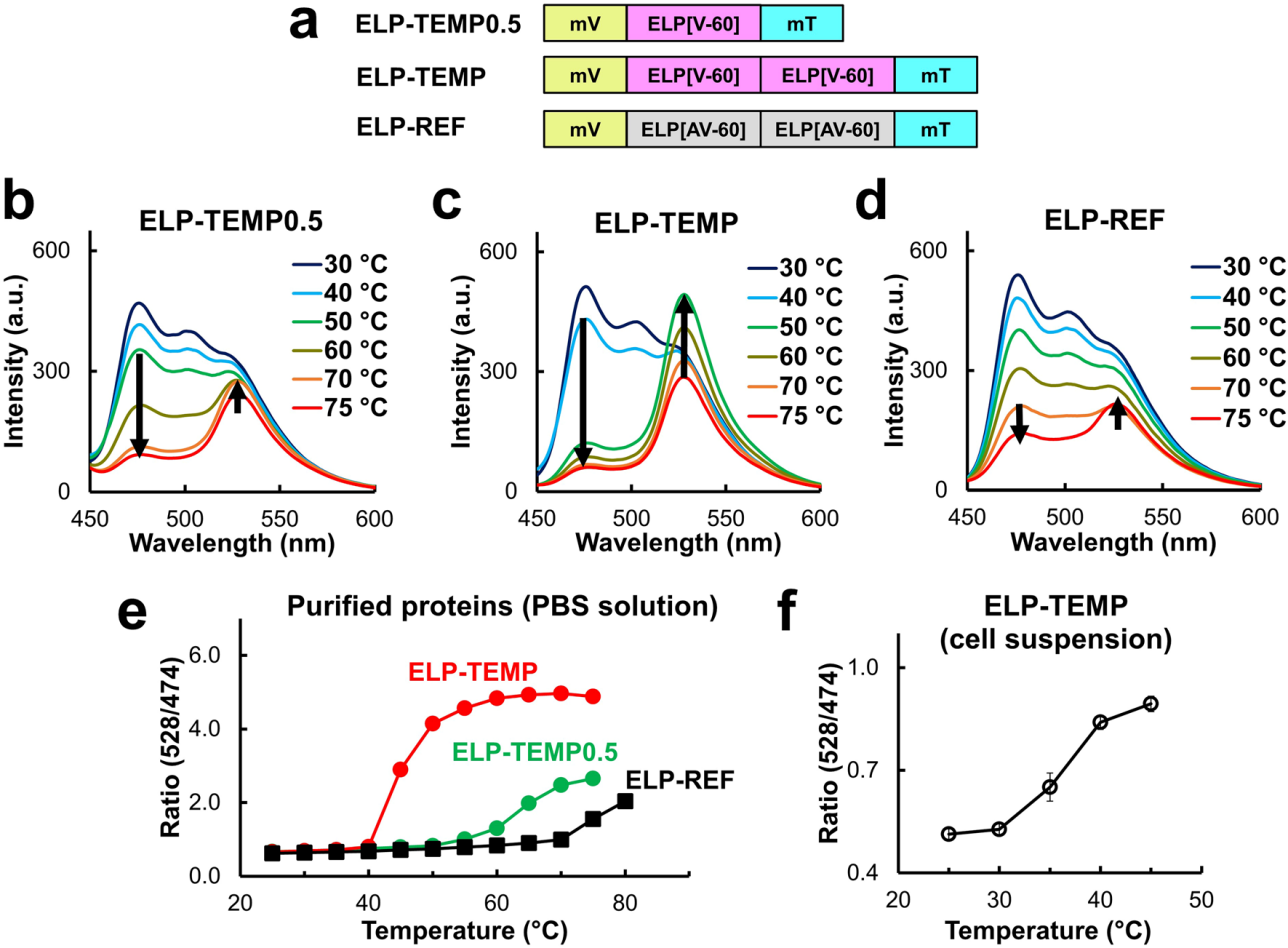
Temperature response of ELP-TEMP0.5, ELP-TEMP, and ELP-reference (ELP-REF). (**a**) Gene design of ELP-TEMP0.5, ELP-TEMP, and ELP-REF. (**b**–**d**) The fluorescence emission spectrum of (**b**) ELP-TEMP0.5, (**c**) ELP-TEMP, and (**d**) ELP-REF at various temperatures. (**e**) A plot of fluorescence ratio of mV (peak emission, 528 nm) to mT (peak emission, 474 nm) of ELP-TEMP0.5, ELP-TEMP, and ELP-REF against temperature. (**f**) A plot of fluorescence ratio of ELP-TEMP in HeLa cell suspension against temperature. Purified proteins (2 µM) were dissolved in a PBS solution (pH 7.4). The excitation wavelength was 430 nm. Data are mean ± SD (*n* = 3).

We exploited the LCST behavior of ELPs to develop a FRET-based GETI. We designed our GETI so that it contained an ELP fused with a FRET pair of mT and mV. For the purpose of temperature imaging in mammalian cells, we selected ELP[V-60] containing a sequence of (VPGVG)_60_, whose *T*_t_ was reported to be 35.2 °C^30^. Thereby, we constructed the gene of a fusion protein of mV-ELP[V-60]-mT denoted as ELP-TEMP0.5, an ELP-based temperature indicator (Fig. 1a). The fluorescence emission spectrum of ELP-TEMP0.5 excited at 430 nm in a phosphate buffered saline solution (PBS) showed fluorescence emission bands with peaks at 474 and 528 nm, which are attributed to mT and mV, respectively (Fig. 1b). When we measured the fluorescence response of ELP-TEMP0.5 by scanning the temperature, the emission peak intensity of mT dramatically decreased as the temperature changed from 50 to 75 °C, whereas that of mV increased (Fig. 1b). Figure 1e shows a plot of the fluorescence ratio of the fluorescence peak intensity of mV to that of mT (528/474 nm) against temperature. The data showed that as the temperature increased from 50 °C, the fluorescence ratio abruptly increased to a higher value, which was most likely ascribable to the phase transition behavior of the ELP (see discussion). Unfortunately, this temperature response was too high for mammalian cell imaging, and thereby, we decided to develop an ELP-based GETI with a lower temperature response. Because an ELP with a large number of the pentapeptide repeats is known to show a low *T*_t_^31^, we then created a fusion protein consisting of a doubly repeated ELP[V-60], mT and mV, denoted as ELP-TEMP (Fig. 1a). ELP-TEMP also showed two fluorescence bands due to mT and mV like ELP-TEMP0.5 (Fig. 1c), but the temperature response was found to shift to a considerably lower temperature around 40–55 °C (Fig. 1e). Although this temperature response as measured in the PBS solution seemed somewhat higher than the optimum for temperature imaging in mammalian cells, the fluorescence ratio measured from a suspension of HeLa cells expressing ELP-TEMP was found to exhibit the transition of fluorescence ratio in the range of 30–45 °C (Fig. 1f), which was optimal for imaging of mammalian cells. Thus, ELP-TEMP is likely to be useful as a GETI for live mammalian cells.

As a reference, we constructed another ELP-FP fusion protein, denoted as ELP-REF, composed of mT, mV, and an ELP with a doubly repeated sequence of ELP[AV-60] ((VPGAG-VPGVG)_30_), which had the same polypeptide length with that of the ELP-TEMP (Fig. 1a). Because ELP[AV-60] itself was known to transition at 55.2 °C^30^, which was substantially higher than *T*_t_ of ELP[V-60]^30^, ELP-REF was expected to transition at a temperature higher than *T*_t_ of ELP-TEMP. In fact, *T*_t_ of ELP-REF was above 70 °C and the fluorescence ratio was almost unchanged between 25 and 60 °C (Fig. 1d,e), regardless of the temperature dependence of the fluorescence intensities of mT and mV (Fig. S1).

### In vitro characterization of ELP-TEMP

**Figure 2:**
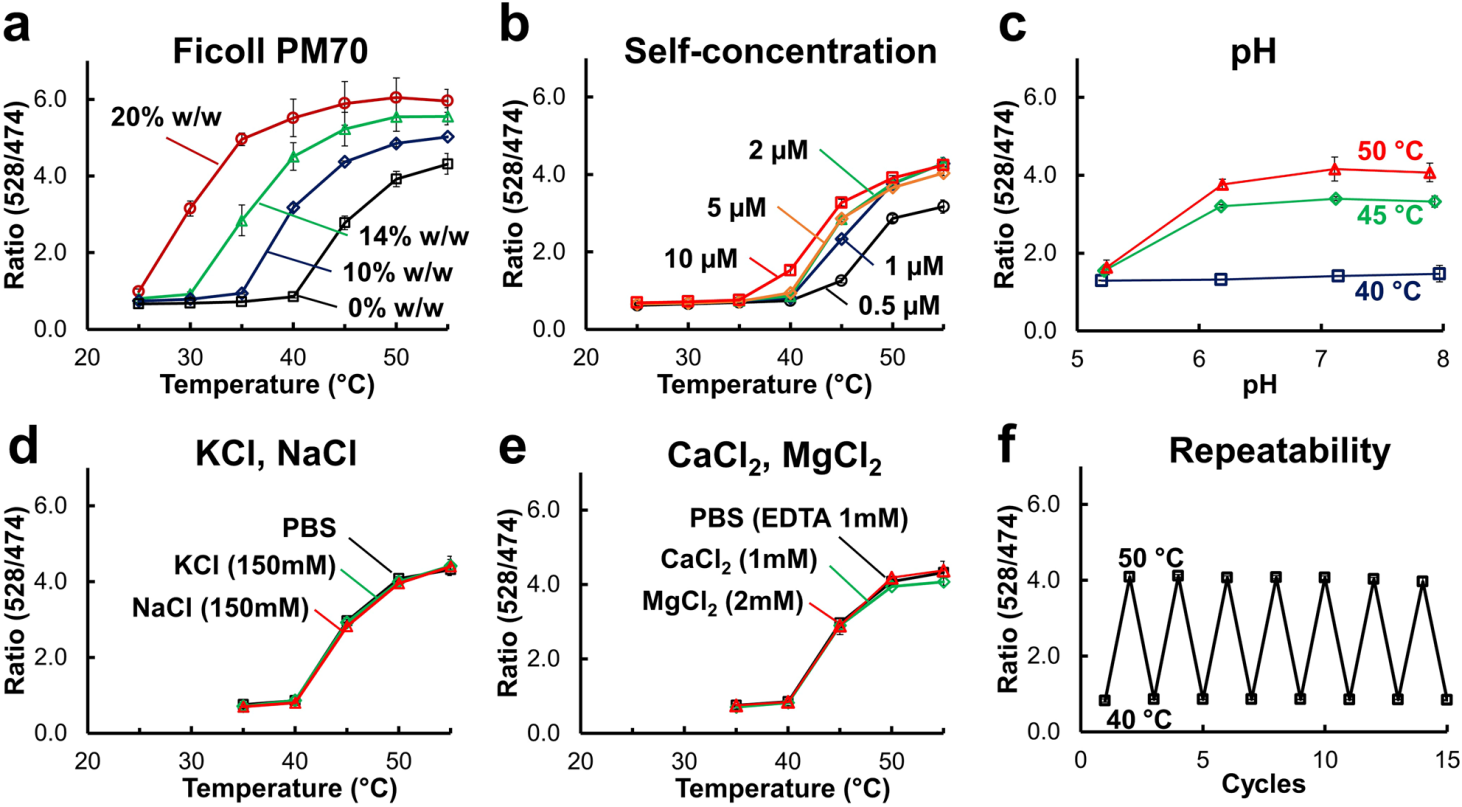
Fluorescence response of ELP-TEMP to temperature in various factors. (**a**) The effect of Ficoll PM70 as a macromolecular crowding reagent on the temperature dependence of fluorescence ratio mV/mT (528/474 nm) of ELP-TEMP. Purified proteins were dissolved in PBS solution containing Ficoll PM70 (0, 10, 14, and 20% w/w; pH 7.4). (**b**) The effect of self-concentration of ELP-TEMP on its temperature dependence of fluorescence ratio. (**c**) The pH-dependence of ELP-TEMP fluorescence ratio. The ELP-TEMP solution contained 30 mM trisodium citrate and 30 mM borax, whose pH was adjusted by adding HCl. The pH values of the solution were directly measured at various temperatures. (**d**) The effect of KCl or NaCl on the temperature response of ELP-TEMP. The ELP-TEMP solution contained PBS solution or 10 mM sodium phosphate buffer with 150 mM KCl or NaCl, pH 7.4. (**e**) The effect of CaCl_2_ or MgCl_2_ on the temperature response of ELP-TEMP. The ELP-TEMP solution contained PBS solution and one of the salts. To mimic 0 mM of CaCl_2_ or MgCl_2_, we added EDTA to the final concentration of 1 mM to the PBS solution. (**f**) The effect of repeatability on the florescence ratio of ELP-TEMP. The protein concentration was 2 µM for all measurements unless otherwise stated. The excitation was 430 nm. Data are mean ± SD (*n* = 3).

We examined the effect of macromolecular crowding, self-concentration, and ion species on the temperature response of ELP-TEMP, as well as the reversibility of temperature response (Fig. 2). To investigate the effect of macromolecular crowding, we utilized Ficoll PM70 as a macromolecular crowding reagent, because it is widely-used as a standard reagent that mimics the intracellular macromolecular crowding^27^. Although the ELP-TEMP fluorescence ratio also responded to elevated temperature in the presence of Ficoll PM70, the transition temperature shifted to lower temperatures as the Ficoll PM70 concentration increased (Fig. 2a), indicating that the temperature response of ELP-TEMP was affected by macromolecular crowding. At 14% w/w Ficoll PM70, the temperature response range of ELP-TEMP was 30–45 °C, which was almost consistent with that in cell suspension (Fig. 1f), suggesting that the macromolecular crowding in HeLa cells would be equivalent to that in a PBS solution containing 14% w/w Ficoll PM70. The temperature response of ELP-TEMP was also dependent to some degree on the self-concentration: the transition temperature shifted to lower temperatures as the ELP-TEMP concentration increased (Fig. 2b). The pH-dependence of ELP-TEMP fluorescence showed that the fluorescence ratio at 40 °C was almost unchanged between pH 6 and 8, but that at 45 and 50 °C declined below pH 6 (Fig. 2c). The temperature response of ELP-TEMP was little affected by the addition of KCl, NaCl, MgCl_2_, or CaCl_2_ (Fig. 2d,e). Additionally, the fluorescence ratio of ELP-TEMP responded to temperature change highly reversibly during many cycles of heating and cooling (Fig. 2f).

### Temperature response of ELP-TEMP in live HeLa cells

**Figure 3:**
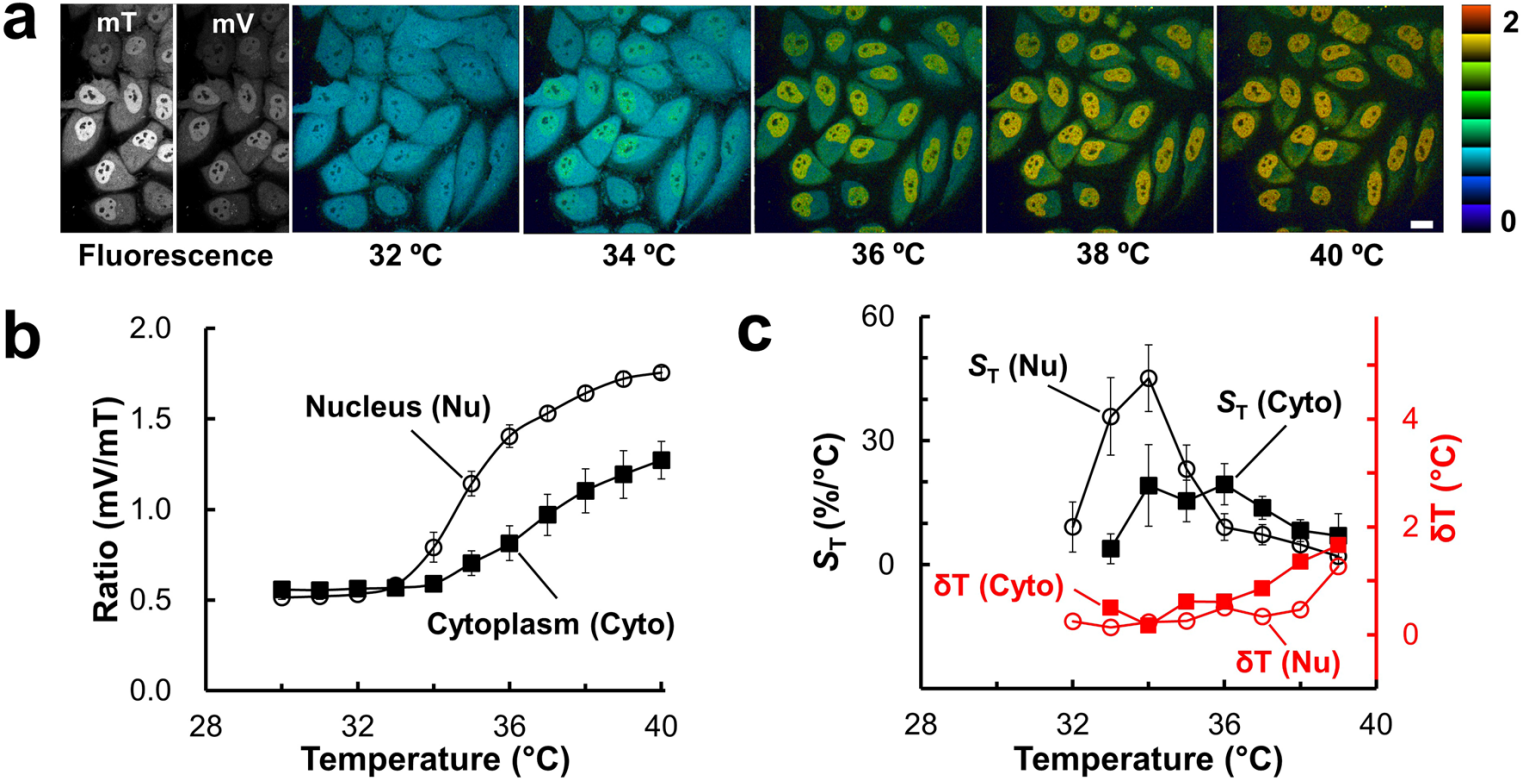
Temperature response of ELP-TEMP stably expressed in live HeLa cells under confocal microscopy observation. (**a**) Confocal fluorescence images and pseudo-colored ratio images of HeLa cells stably expressing ELP-TEMP at various temperatures. (**b**) A plot of fluorescence ratio mV/mT in the nucleus (Nu) and cytoplasm (Cyto) against medium temperature. (**c**) A plot of relative temperature sensitivity (*S*_T_; left vertical axis) and temperature resolution (δT; right vertical axis) of the Nu or Cyto against medium temperature. The color bar indicates fluorescence ratio mV/mT. Scale bar, 20 µm. Data are mean ± SD (*n* = 18).

We investigated the temperature response of ELP-TEMP in live HeLa cells. When we observed HeLa cells stably expressing ELP-TEMP by a confocal microscope, we were able to capture the fluorescence of mT and mV from the nucleus and cytoplasm in each cell, indicating that ELP-TEMP was present in both (Fig. 3a). By analogy with the spectroscopy characterization of ELP-TEMP (Fig. 1), we derived ratio images of the fluorescence of mV to that of mT (mV/mT) at various temperatures to examine the temperature response of ELP-TEMP in the cells, confirming that the fluorescence ratio changed with temperature between 34–40 °C (Fig. 3a). By looking at the nucleus and cytoplasm, we noticed that the fluorescence ratios responded to temperature somewhat differently between them (Fig. 3a). Thus, we separately analyzed the temperature-dependent fluorescence ratios in the nucleus and cytoplasm (Fig. 3b). The data showed that the phase transition temperature in the nucleus would be lower by 1.5 °C than that in the cytoplasm (see Methods for detail), and the fluorescence ratio in the nucleus was significantly higher than that in the cytoplasm. These differences would be mainly attributed to a difference in ELP-TEMP concentration (see Investigation of the temperature in the nucleus and cytoplasm with ELP-TEMP). Furthermore, we examined a relative temperature sensitivity *S*_T_ defined as,

1 $$S_{{\text{T}}} = \frac{\Delta R}{R} \cdot \frac{1}{\Delta T} \times 100 (\%\;^{\circ} {\text {C}}),$$

where *R* is a fluorescence ratio mV/mT, *T* is temperature, and ∆ denotes a change of a parameter value with changing temperature. As shown in Fig. 3c, *S*_T_ of ELP-TEMP showed values of 5–45%/°C between 32 and 40 °C and maxima of 45.1 ± 8.1%/°C at 34 °C and 19.5 ± 5.0%/°C at 36 °C in the nucleus and cytoplasm, respectively. Compared from *S*_T_ values of previous GETIs (tsGFP1, *S*_T_ = 3.9%/°C^14^; gTEMP, *S*_T_ = 2.6%/°C^2^; emGFP-mito, *S*_T_ = 2.2%/°C^32^; EGFP, *S*_T_ = 2.1%/°C^33^; EGFP in fluorescence polarization anisotropy, *S*_T_ = 0.5%/°C^34^) and non-genetically-encoded indicators for intracellular thermometry (Table S1 in the Supplementary Information), the present *S*_T_ of ELP-TEMP would be the highest ever reported temperature sensitivity. In addition, we evaluated a temperature resolution δ*T* defined as,

2 $$\delta T = \frac{\Delta T}{{\Delta R}} \cdot \sigma (R)  ({^{\circ}}\text{C}),$$

where *σ*(*R*) represents the standard deviation of the fluorescence ratio. The δ*T* value of ELP-TEMP was as fine as < 1 °C between 33 and 37 °C with the finest δ*T* of 0.1 °C at 33 °C (Fig. 3c). In contrast, when we observed live HeLa cells stably expressing ELP-REF, we detected only a very small change in fluorescence ratios from 30 to 40 °C (*S*_T_ = 0–3.7%/°C) (Fig. S2).

It should be noted that we used a HeLa cell line with stable ELP-TEMP expression. In fact, HeLa cells with transient ELP-TEMP expression showed a large variety of fluorescence ratios mV/mT (Fig. S3), and thus, they were not suitable for temperature imaging in this study. Taking the data in Fig. 2b into consideration, the variety of fluorescence ratio suggests that, in cells transiently expressing ELP-TEMP, the expression level of ELP-TEMP would considerably differ from cell to cell (Fig. S3c). However, establishing a stable cell line could mitigate this effect as we found that the stable cell line (Fig. 3b) showed less variance in ratios than that of the transient cell line (Fig. S3). Thus, we used the HeLa cell line stably expressing ELP-TEMP throughout the study, unless mentioned.

We examined intracellular macromolecular crowding in HeLa cells, since fluorescence ratio of ELP-TEMP was affected by macromolecular crowding (Fig. 2a). We observed HeLa cell expressing a genetically encoded crowing indicator, crGE^27^, at various temperatures with the same imaging conditions to those of the observation of ELP-TEMP. The fluorescence ratio of crGE was almost independent on temperature from 30 to 40 °C (Fig. S4), indicating that the degree of macromolecular crowding in HeLa cells is likely to be almost unchanged during the microscopy observation. As a reference, crGE was reported to show a fluorescence ratio (mCitrine/mCerulean) change of ~ 16% when the Ficoll PM70 concentration changed from 10 to 20% w/w^27^. This result, as a control, suggests that the temperature response of ELP-TEMP (Fig. 3b) was mostly attributed to temperature change.

### Application of ELP-TEMP for measuring temperature changes in live HeLa cells

**Figure 4:**
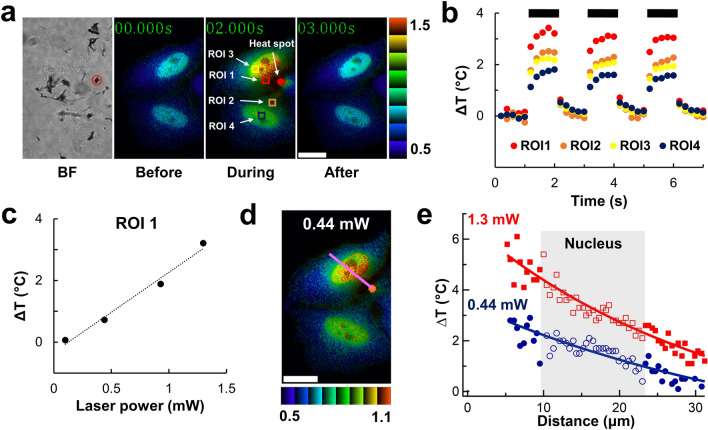
Application of ELP-TEMP to monitor quick temperature rise in live HeLa cells with a local heat spot. (**a**) Bright-field (BF) and pseudo-colored ratio images of HeLa cells stably expressing ELP-TEMP before, during, and after local heating. The red circle indicates the CNT cluster, i.e., the heat spot, whereas squares indicate regions of interest (ROI). (**b**) A plot of temperature increment Δ*T* as the function of time in ROIs 1–4. The temperature of ROI 1 and 2 was estimated from the cytoplasm calibration curve, whereas the temperature of ROI 3 and 4 was estimated from the nucleus calibration curve in Fig. S5, respectively. A CNT cluster located near two cells was irradiated with a 638 nm laser beam at a power of 1.3 mW. Black lines indicate the periods of laser irradiation. (**c**) A plot of Δ*T* in ROI 1 as a function of the laser power at 638 nm. (**d**) A pseudo-colored ratio image of HeLa cells stably expressing ELP-TEMP under local heating with the laser power of 0.44 mW. A line indicates ROI. (**e**) A plot of Δ*T* of the line in panel (**d**) against the distance from the heat spot. Closed and opened blue circles represent Δ*T* in the cytoplasm and nucleus, respectively, with the laser power of 0.44 mW, whereas closed and opened red squares represent Δ*T* in the cytoplasm and nucleus, respectively, with the laser power of 1.3 mW. The transparent box indicates the nucleus area. The medium temperature was 34 °C. The color bar indicates fluorescence ratio mV/mT. Scale bars, 20 μm.

We employed ELP-TEMP to demonstrate the imaging of quick temperature rises in live HeLa cells. We observed HeLa cells stably expressing ELP-TEMP mixed with a suspension of carbon nanotubes (CNTs) on an in-house built fluorescence microscope. We chose a CNT cluster near to two HeLa cells in the culture medium and irradiated it with a focused laser beam at 638 nm (Fig. 4a). The laser beam irradiation brought about local heat production in the CNT cluster followed by heat diffusion from it^35^. In fact, when we turned on the beam irradiation of the CNT cluster, an increase of fluorescence ratio in the cells was observed, indicating a temperature increase (Fig. 4a and Supplementary movie 1). When we turned off the beam irradiation, the fluorescence ratio decreased to the original level, indicating a temperature decrease, due to heat dissipation. Furthermore, we used the calibration of fluorescence ratio as a function of temperature taken separately from the nucleus and cytoplasm (Fig. S5) to determine the temperature increment ∆*T* in regions of interests (ROIs) 1–4 relative to the surrounding medium temperature (Fig. 4a). A plot of ∆*T* against time (Fig. 4b) shows that the read-out of temperatures from ELP-TEMP reversibly responded to the laser irradiation of the CNT cluster. Additionally, the dependence of the plateau ∆*T* in ROI 1 during heating on the laser power (Fig. 4c) confirmed that the increase of ∆*T* was well correlated with the energy flux directing the CNT cluster. Thus, these data demonstrate that we successfully applied ELP-TEMP to detect reversible temperature changes due to the heat production and dissipation arising from turning on and off the heat production in the CNT cluster.

We examined a cross-section of ∆*T* during heating across the nucleus and cytoplasm in a HeLa cell. We took a profile of the plateau temperature increment ∆*T* during heating along a line which crossed the CNT cluster, i.e., the heat spot, the nucleus, and the cytoplasm (Fig. 4d,e). It should be noted that because the calibrations of the fluorescence ratios as a function of temperature were significantly different between the nucleus and cytoplasm (Fig. S5), we calculated ∆*T* values in them separately using the corresponding calibrations. A plot of ∆*T* as a function of the distance from the heat spot shows that the values of Δ*T* depended on both the distance from the heat spot and the total laser power at 638 nm. Furthermore, we found that the Δ*T* values seemed to continuously decrease with the distance irrespective of whether a ROI was in the nucleus or cytoplasm, and thus, a discontinuity in ∆*T* seemed not to be noticeable at the interface between them (Fig. 4e). The fluorescence intensity measured from a pixel in Fig. 4d should reflect not only the fluorescence at the corresponding in-focus region on the specimen plane but also one from its surroundings. Considering that we observed the cells (thickness, ~ 16 μm) by wide-field fluorescence microscopy, we estimated that the distance from the CNT cluster to a position on the in-focus plane has an uncertainty of 5.8–7.5 μm (Fig. S6), meaning that the distance-temperature profile (Fig. 4e) could be smoothed to some degree. However, it is with the use of the corresponding calibrations (Fig. S5) that we were able to determine the temperatures separately in the nucleus and cytoplasm to find the continuity of the distance-temperature profile across them. Thus, we suggest that within the present measurement precision there seemed no detectable temperature gap along the line we investigated. Additionally, because of the high *S*_T_, ELP-TEMP was able to map a temperature difference in single cells even if Δ*T* was as small as < 1 °C (Fig. 4d). These results altogether demonstrate that ELP-TEMP would be a very useful GETI to visualize intracellular temperature dynamics that have been previously invisible on the micrometer to nanometer scale.

**Figure 5:**
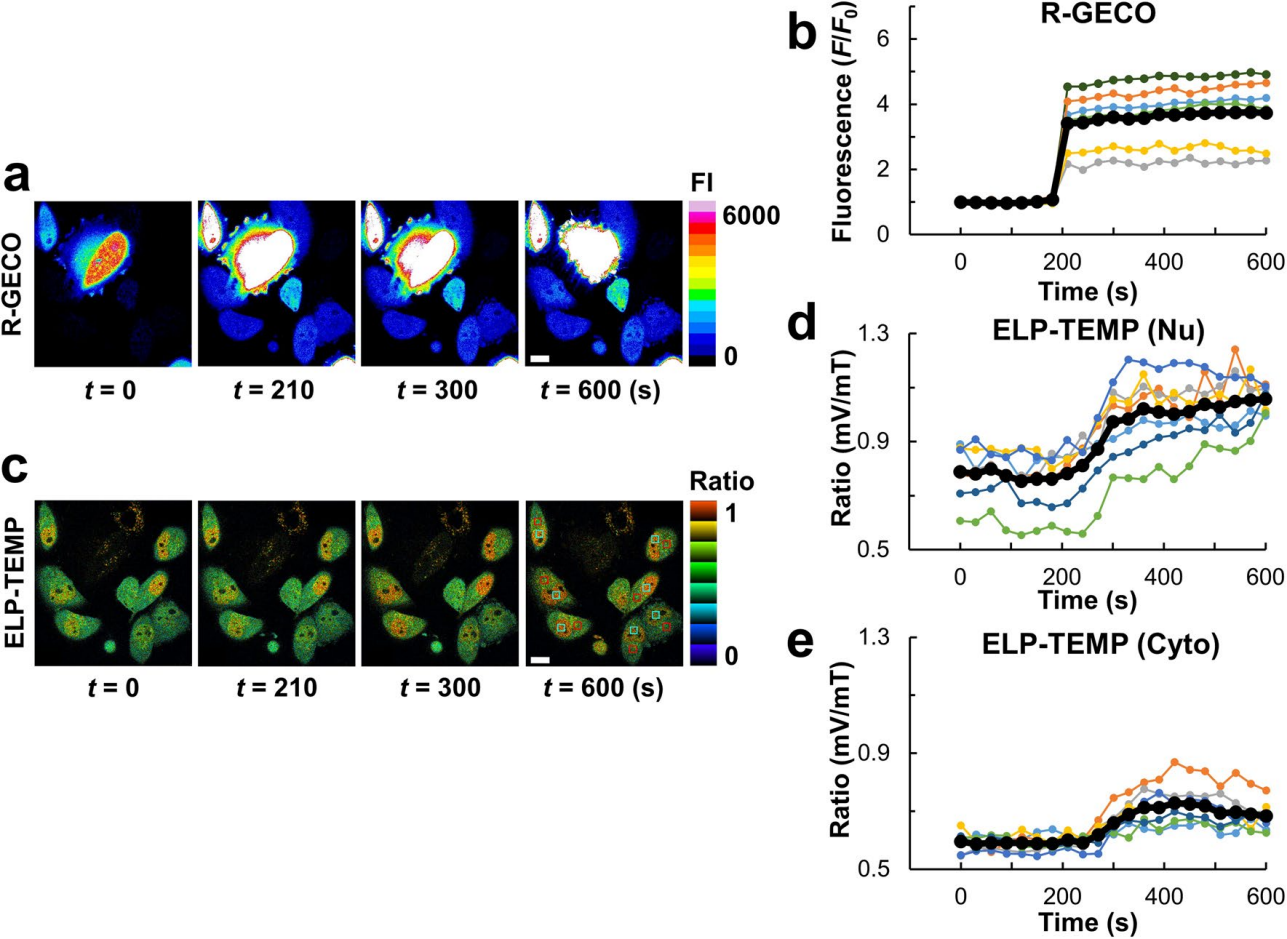
Visualization of heat production by stimulated Ca^2+^ influx with a Ca^2+^-ionophore ionomycin in live HeLa cells stably expressing ELP-TEMP and transiently co-expressing R-GECO. (**a**) Pseudo-colored fluorescence images of R-GECO in response to ionomycin stimulation. (**b**) A plot of fluorescence intensity (*F*/*F*_0_) of R-GECO against time. (**c**) Pseudo-colored ratio images of ELP-TEMP in response to ionomycin stimulation. (**d**) A plot of fluorescence ratio mV/mT of ELP-TEMP in the nuclues (Nu) against time. (**e**) A plot of fluorescence ratio mV/mT of ELP-TEMP in the cytoplasm (Cyto) against time. The observation was performed under the same confocal micoscope in Fig. 3. To prevent changes in medium temperature due to ionomycin stimulation, medium containing 4 µM ionomycin was supplied through a preheated perfusion tube, and the medium temperature around the observed cells was maintained at 34 ± 0.1 °C during observation. Red and cyan squares indicate ROIs for cytoplasm and nuclues, respectively. The color bars indicate fluorescence intensity (FI) and ratio for (**a**) and (**c**), respectively. Scale bars, 20 µm.

We tried to detect heat production caused by ionomycin-induced Ca^2+^ influx, which has been reported to give rise to an intracellular temperature increase by 1–2 °C^10,11,36^. We stimulated HeLa cells stably expressing ELP-TEMP with 4 µM ionomycin by perfusion, and observed the cells with the same confocal microscope as that in Fig. 3 so that we could determine the intracellular temperature from the calibration in Fig. 3b. We transiently co-transfected the HeLa cells stably expressing ELP-TEMP with a genetically encoded Ca^2+^ indicator, R-GECO^37^, to monitor Ca^2+^ concentration change. As shown in Fig. 5a,b and Supplementary movie 2, we detected an abrupt increase of Ca^2+^ concentration at *t* = 210 s in the cells through R-GECO fluorescence, being most likely due to Ca^2+^ influx stimulated by ionomycin. Furthermore, we found that the Ca^2+^ concentration increase was followed by a significant increase of the fluorescence ratio mV/mT of ELP-TEMP in both nucleus and cytoplasm (two-tailed Student’s *t-*test led to *p* = 0.0007 and 0.007 for nucleus and cytoplasm, respectively, between *t* = 0 and *t* = 600 s in Fig. 5d,e). As a control, we also took the fluorescence image of mV directly-excited at 514 nm during the observation of Fig. 5 as a measure of ELP-TEMP average concentration in the ROIs (see Investigation of the temperature in the nucleus and cytoplasm with ELP-TEMP). The fluorescence intensity of directly-excited mV was observed to be almost unchanged with the Ca^2+^ concentration increase induced by ionomycin stimulation, and thus, the ELP-TEMP concentration would have been almost unchanged during this observation (Fig. S7). As another control, we examined macromolecular crowding in HeLa cells transfected with both macromolecular crowding indicator crGE^27^ and R-GECO before and after ionomycin stimulation. However, the fluorescence ratio mCitrine/mCerulean of crGE, i.e., macromolecular crowding was unchanged (Fig. S8c,d; Supplementary movie 3) even though Ca^2+^ concentration increased as observed by R-GECO fluorescence increase (Fig. S8a,b; Supplementary movie 3). In addition, we should also note that the fluorescence ratio of ELP-TEMP was little affected by the addition of CaCl_2_ (Fig. 2e). Accordingly, considering the results thus far, the fluorescence ratio increase from ELP-TEMP after ionomycin stimulation (Fig. 5d,e) is likely to be attributed to temperature increase caused by Ca^2+^ influx. Using the nucleus and cytoplasm calibration curves in Fig. 3b, we were able to estimate the average temperature increase (∆*T*) before (average temperature in a range of *t* = 0–180 s) and after ionomycin stimulation (average temperature in a range of *t* = 420–600 s) in the nucleus and cytoplasm, respectively, to be ∆*T* = 1.0 ± 0.7 °C and 1.5 ± 0.8 °C (means ± SD, *n* = 7), which was consistent with previously reported studies^10,11,36^.

### Investigation of the temperature in the nucleus and cytoplasm with ELP-TEMP

**Figure 6:**
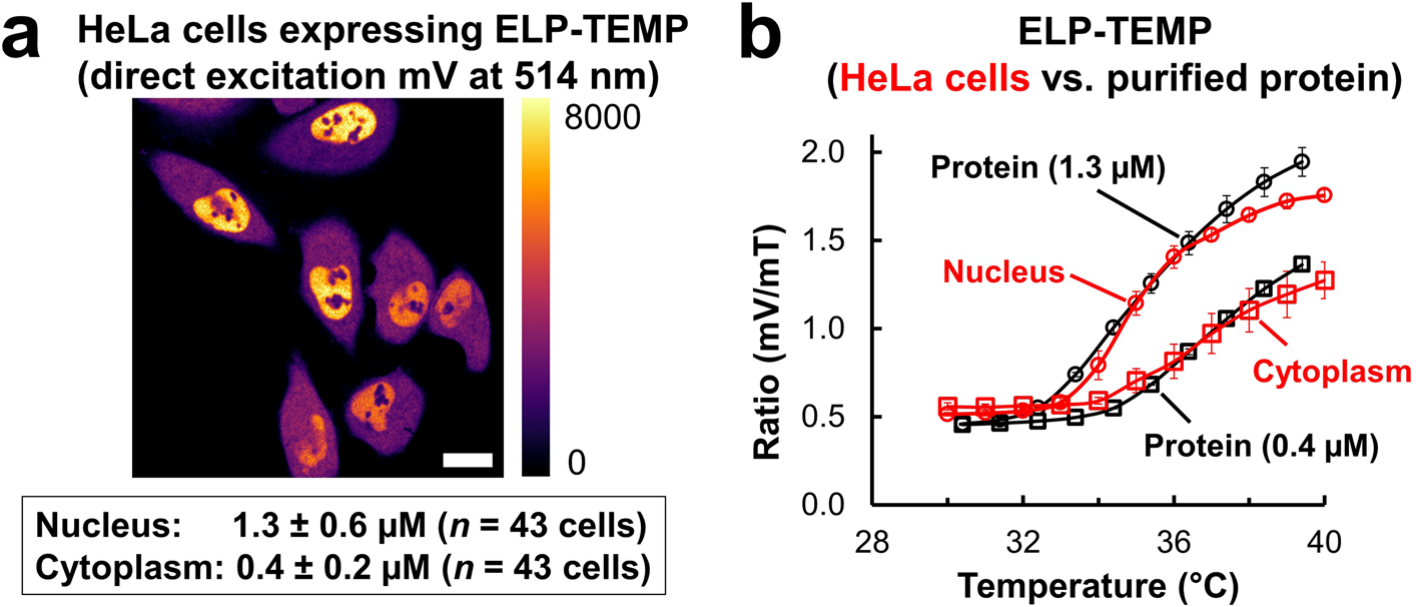
Investigation of the difference in fluorescence ratios of ELP-TEMP between the nucleus and cytoplasm. (**a**) Fluorescence image of mV signal in ELP-TEMP by direct excitation at 514 nm observed by a confocal microscope. The mV fluorescence intensity in the nucleus was measured to be 3.3-fold higher than that in the cytoplasm. From the calibration curve (Fig. S9), the ELP-TEMP concentration in the nucleus and cytoplasm was estimated to be 1.3 ± 0.6 µM and 0.4 ± 0.2 µM (*n* = 43 cells), respectively. The color bar indicates fluorescence intensity of mV. Scale bar, 20 μm. (**b**) Comparison of temperature response of ELP-TEMP in HeLa cells measured by confocal microscopy (Fig. 3b) with that of purified protein at concentrations of 1.3 and 0.4 µM in a PBS solution containing 14% w/w Ficoll PM70 by fluorescence spectroscopy. The correlation coefficients between the fluorescence ratios from cells and purified protein were 0.995 and 0.999 for nucleus and cytoplasm, respectively, where the data points on the trajectories of purified protein were interpolated by spline curves. For comparison with the microscopy data, we calculated the integral of *F*(*λ*)*T*(*λ*) for mT and mV, where *F* is a fluorescence emission intensity at a wavelength *λ* and *T* is a composite spectral transmittance of a bandpass filter and a dichroic mirror. The integration wavelengths were 457–500 nm and 526–552 nm for mT and mV, respectively. Additionally, we directly measured the temperature of the ELP-TEMP solution with the same thermometer used for the microscopy observation. The excitation was 430 nm. Data are mean ± SD (*n* = 3).

We investigated the difference in the temperature response of ELP-TEMP between the nucleus and cytoplasm observed in Fig. 3a,b. Since we clearly observed a difference in ELP-TEMP fluorescence intensities between the nucleus and cytoplasm (Fig. 3a), we speculated that the ELP-TEMP concentration may have been different between them. Thus, we tried to measure ELP-TEMP concentrations in the nucleus and cytoplasm to figure out the accurate temperature response of ELP-TEMP in them. To measure the ELP-TEMP concentration in cells, we measured the mV fluorescence in ELP-TEMP by direct excitation of mV at 514 nm with a confocal microscope. Because mV is the acceptor of FRET in ELP-TEMP, its fluorescence intensity by direct excitation should not be susceptible to the change of FRET efficiency, and therefore, it was expected to be little dependent on the conformation of the ELP moiety. As shown in Fig. 6a, the mV fluorescence intensity in the nucleus was measured to be 3.3-fold higher than that in the cytoplasm, and thereby, ELP-TEMP concentrations in the nucleus and cytoplasm were estimated as 1.3 ± 0.6 µM and 0.4 ± 0.2 µM, respectively, by using a calibration of the mV fluorescence intensity by direct excitation from purified ELP-TEMP (Fig. S9). In addition, since a result from Western Blotting showed that ELP-TEMP was little degraded in HeLa cells (Fig. S10), the mV fluorescence intensity from the cells in Fig. 6a was mostly ascribed to ELP-TEMP. With the information of ELP-TEMP concentrations, we then measured the temperature response of purified ELP-TEMP in a PBS solution at concentrations of 1.3 and 0.4 μM corresponding to ELP-TEMP concentrations in the nucleus and cytoplasm, respectively, and compared their temperature responses with those measured in cells (Fig. 6b). Note that to mimic the intracellular macromolecular crowding, we added 14% w/w Ficoll PM70 to the PBS solution, because this Ficoll PM70 concentration well emulated the temperature response of 1.3 µM ELP-TEMP in the nucleus (Fig. S11). As shown in Fig. 6b, the temperature response of ELP-TEMP at concentrations of 1.3 and 0.4 µM in the presence of 14% w/w Ficoll PM70 was highly consistent with the temperature response of ELP-TEMP in the nucleus and cytoplasm, respectively. Furthermore, we also examined the macromolecular crowding in the nucleus and cytoplasm on the temperature response of ELP-TEMP. When we observed HeLa cells expressing the macromolecular crowding indicator of crGE^27^, we found that the fluorescence signals from the nucleus and cytoplasm were almost the same between 30 and 40 °C (Fig. S4), indicating that the macromolecular crowding was almost equivalent in them. This result suggests that macromolecular crowding would have very little effect on the observed difference of ELP-TEMP temperature response between the nucleus and cytoplasm. Thus, considering the results presented here, the difference in the temperature response of ELP-TEMP between the nucleus and cytoplasm would be mainly attributed to the ELP-TEMP concentration difference in them.

## Discussion

In this study, we successfully developed a highly-sensitive GETI, ELP-TEMP, exploiting the LCST behavior of an ELP fused with a FRET pair of FPs for intracellular temperature measurement in mammalian cells with high accuracy. Because ELP-TEMP achieved the highest ever *S*_T_ among reported fluorescent nanothermometers, ELP-TEMP would solve the main drawback of poor *S*_T_ of GETIs. In addition, because the temperature imaging with ELP-TEMP provided a quantitative measurement and ELP-TEMP was expressed in live cells by simply transfecting the cells with a common transfection method, ELP-TEMP has overcome the limitation of other approaches using ELPs for temperature measurement such as ELPs fused with a GFP that only detect a temperature at the *T*_t_ of ELPs^19^ and ELP chemically labeling with a hydrophobicity-sensitive fluorescent dye^20^. We found that ELP-TEMP showed sensitivity not only to temperature but also to macromolecular crowding and self-concentration. However, by the observation with a genetically encoded crowding indicator, crGE^27^, the possibility of the effect of the macromolecular crowding on the temperature response of ELP-TEMP was ruled out as far as the imaging conditions in the present study applied. Additionally, by using the stable cell line, we mitigated the effect of different expression levels of ELP-TEMP, i.e., different intracellular ELP-TEMP concentrations, from cell to cell, and by measuring the concentrations of ELP-TEMP in the nucleus and cytoplasm separately through the fluorescence of directly excited mV, we were able to determine the accurate temperature response of ELP-TEMP in them, which depended on ELP-TEMP concentration. Furthermore, we showed that, by changing the polypeptide length of the ELP moiety and the guest residue in the pentapeptide, the temperature response of ELP-TEMP can be optimized to fit the physiological temperature of mammalian cells. Thus, by further modifying the length of ELP moiety, a new version of ELP-TEMP with a different temperature response range can be developed for a wide range of different applications such as temperature imaging in medaka fish, whose optimum temperature for rearing was reported at 28 °C^38^, or some plants growing with an optimum temperature range of 20–30 °C^39^.

The fluorescence ratio of ELP-TEMP changed in response to the temperature in relation with FRET. Previously, Tang et al*.* reported *T*_t_ of ELPs themselves as determined by solution turbidity by measuring the absorbance at 350 nm, and the order of *T*_t_ was ELP[AV-60] > ELP[V-60] > ELP[V-150]^30^. The temperature response *T*_t_ of our ELP-FP fusion proteins showed an order of ELP-REF (ELP[AV-60]-ELP[AV-60]) > ELP-TEMP0.5 (ELP[V-60]) > ELP-TEMP (ELP[V-60]-ELP[V-60]). In the standpoint of the guest residues in the pentapeptide, the order of ELP[AV-60] > ELP[V-60] should be consistent with that of ELP-REF > ELP-TEMP, which had the same polypeptide length. In the standpoint of the length of ELP moiety, the order of ELP[V-60] > ELP[V-150] should be consistent with that of ELP-TEMP0.5 > ELP-TEMP, which had the same guest residue. Thus, these results suggest that the transitions of fluorescence ratio seen in Fig. 1e should be largely attributed to the change of ELP moiety. Li et al*.* investigated the temperature-dependent conformation of ELPs by molecular dynamics simulation and showed that at low temperature, the ELP molecules were in a much more extended conformation, whereas at temperatures above the *T*_t_, ELP backbones underwent conformation changes and formed a highly compacted coacervate^40^. Thus, it is suggested that below *T*_t_, the conformation of ELP-FP fusion proteins, i.e., ELP-TEMP0.5, ELP-TEMP, or ELP-REF would be largely extended and dispersed^17^ in the solution so that FRET from mT to mV occurs at a low efficiency. At temperatures above *T*_t_, the ELP moiety in the ELP-FP fusion proteins would undergo the conformation change to form the compacted coacervate so that the average distance between mT and mV would become smaller than that below *T*_t_, and thus, FRET between them occurs at a high efficiency (Fig. S12). In fact, at low temperatures, we observed a high mT fluorescence and a low mV fluorescence, indicating low FRET efficiency, whereas at elevated temperatures above the *T*_t_, the mT fluorescence was abruptly decreased accompanied with an increase of mV fluorescence, indicating high FRET efficiency. This indicates that FRET efficiency is changed in accordance with the phase transient behavior of the ELPs, which is consistent with our hypothesis. Thus, ELP-TEMP would be the first FRET-based GETI, to our knowledge.

It should also be noted that the *T*_t_ of ELP-TEMP0.5 was significantly higher than that of the ELP[V-60] polypeptide itself^30^. This difference has been known as the “fusion ∆*T*_t_ effect” (fusion ∆*T*_t_ = *T*_t_ (ELP fusion protein)−*T*_t_ (free ELP)), which was caused by fusion with proteins that might change the conformation stability and the hydration surrounding the ELP^31,41^. Particularly, fusion with a hydrophobic protein resulted in a decrease of *T*_t_, whereas fusion with a hydrophilic protein resulted in an increase of *T*_t_ of the ELP fusion protein compared to that of the ELP alone^31,41^. In our design, because ELPs are fused with two FPs, which have highly hydrophilic surface^41^, the soluble state of the ELP-FP fusion proteins would be more favorable than that of the unfused ELPs, causing a shift to a higher transition temperature of the ELPs in the fusion proteins than that of the ELPs alone. Thus, the *T*_t_ of ELP-TEMP0.5, ELP-TEMP, and ELP-REF showed higher *T*_t_ than those of unfused ELPs.

We look at the difference of fluorescence ratios of ELP-TEMP in the nucleus and cytoplasm. Although there have been several reports suggesting a temperature difference between the nucleus and cytoplasm in mammalian cells^1–3,42^, it still remains controversial^7–9,43^. According to the results of in vitro characterization, the temperature response of ELP-TEMP was dependent on the self-concentration, macromolecular crowding and the environment pH, and thus, we questioned whether these factors were responsible for the observed difference of fluorescence ratios between the nucleus and cytoplasm. We showed that purified ELP-TEMP at concentrations of 1.3 and 0.4 µM in a PBS solution containing 14% w/w Ficoll PM70 was able to well emulate the temperature response of ELP-TEMP in the nucleus and cytoplasm. In addition, by using crGE, we ruled out the possibility of the effect of macromolecular crowding on the observed difference in the fluorescence ratio of ELP-TEMP. From a previous study, pH in HeLa cells was measured to be 7.4 ± 0.2 pH units without significant difference between the nucleus and cytoplasm^44^. Thus, the difference of the temperature response in the nucleus and cytoplasm should be mainly attributed to the difference of ELP-TEMP concentrations in them. Furthermore, from the result in Fig. 6b, although we assumed that the temperatures in the nucleus and cytoplasm in HeLa cells were the same as that in the culture medium, the fluorescence ratio of purified ELP-TEMP in a temperature control of the fluorescence spectrophotometer was in good agreement with the microscopy data in the nucleus and cytoplasm. The result in Fig. 4e suggested that there seemed no noticeable thermal boundary at the interface between the nucleus and cytoplasm. As shown in Fig. 3a, the fluorescence ratio of ELP-TEMP was observed to be almost uniform in the nucleus or cytoplasm. Considering the evidence presented here together with Fig. 6, the temperatures in the nucleus and cytoplasm in the absence of heat source and any stimulation should be almost the same within the temperature resolution of our measurement.

In the present study, we examined temperature in HeLa cells in the steady state and the drug-stimulated state. In the heating experiment using a CNT cluster and the irradiation of focused laser beam, the temperature in HeLa cells in the steady state changed passively: the temperature changed back and forth rapidly at a time constant of ~ 200 ms with turning on and off the laser beam irradiation (Fig. 4b) and no thermal boundary or gap was noticeable in the distance-temperature profile taken from the cell during heating (Fig. 4e). In addition, the temperatures in the nucleus and cytoplasm of HeLa cells in the steady state without any external heat source and drug stimulation were measured to be indistinguishable (Fig. 6). Baffou et al*.*^7^ estimated by using the heat diffusion equation that the temperature inside a cell could be higher than its surrounding environment by ~ 10^−5^ K, in which they assumed heat conduction in watery environment and a steady-state heat production of ~ 100 pW/cell^7^. The conditions of the cells in Figs. 4 and 6 might be apparently somewhat near to the situation discussed by Baffou et al*.*, considering that the amount of heat production of HeLa cells was measured to be ~ 10^1^ pW/cell^45^ together with the approximation by Baffou et al*.*^7^such that ∆*T* = *P*/*κL*, where ∆*T* is an expected temperature increase (K), *P* is the amount of heat production (W), *κ* is a thermal conductivity (Wm^–1^ K^–1^), and *L* is a typical size of a heat source (m)^7^. In contrast, when HeLa cells were stimulated by the addition of ionomycin, the temperature increase (∆*T* =  ~  1.5 K in the cytoplasm) was observed to last for > 100 s (Fig. 5e). Because ionomycin allows for stimulated transport of Ca^2+^ across a membrane^46^, the influx of Ca^2+^ toward the inside of cells induced by ionomycin occurs as observed by the R-GECO fluorescence (Fig. 5b) and this should lead to the activation of intracellular Ca^2+^ pumps that hydrolyze ATP^47^, possibly having a knock-on effect on the activation of the intricate metabolism. In fact, in the observation of HeLa cells stimulated with ionomycin, the fluorescence response of ELP-TEMP was observed to delay by ~ 120 s after the increase of Ca^2+^ concentration, and this delay has been reported previously^11,36^. Thus, the observation of the continued temperature increase observed in the ionomycin stimulation (Fig. 5d,e) may be compatible with a presumption by Suzuki et al*.*^8^ that an intracellular temperature increase relative to the environment under stimulation could be sustained by continued glucose uptake.

We have demonstrated that, by properly calibrating ELP-TEMP, we are able to measure temperature inside cells for high accuracy at an optical microscopy resolution. Moreover, by localizing ELP-TEMP to a specific organelle by fusing with a targeting peptide or protein, we would be able to measure the temperature in an organelle of interest. Although the fusion of ELP-TEMP with a targeting peptide or protein may change the transition temperature of ELP-TEMP due to the “fusion ∆*T*_t_ effect” of the ELP, it would be possible to be compensated by a proper calibration. Because of the remarkable *S*_T_, we expect that, in the future, ELP-TEMP would be able to reveal small heat production arising from biological processes or drug stimulations that have been previously-invisible at the cellular/subcellular level. Altogether, ELP-TEMP would be a useful GETI for the investigation of cell thermobiology.

## Methods

### Gene construction

We obtained plasmids containing ELP genes from Addgene (ELP[V-60]^30^ containing the sequence of (VPGVG)_60_ , #67013; ELP[AV-60]^30^ containing the sequence (VPGAG-VPGVG)_30_, #67012). To create a doubly repeated ELP[V-60] (ELP[V-60]-ELP[V-60]), we amplified the whole cDNA of ELP[V-60] with both forward and reverse primers containing a *Xho*I restriction enzyme site (*Xho*I-ELP[V-60]-*Xho*I). Meanwhile, we also amplified the cDNA of ELP[V-60] with a forward primer containing a *Sal*I site and a reverse primer containing a *EcoR*I site (*Sal*I-ELP[V-60]-*EcoR*I). We then ligated these two amplified fragments with the *Xho*I-*Sal*I restriction enzyme sites. Because the cut site of *Xho*I (C/TCGAG) and *Sal*I (G/TCGAC) produces compatible cohesive ends, their ligation product can be either GTCGAG or CTCGAC, which cannot be cut by either *Xho*I or *Sal*I restriction enzymes. To create ELP[AV-60]-ELP[AV-60], a similar procedure was conducted. To construct an ELP-TEMP gene, we enzymatically cut the whole cDNA of ELP[V-60]-ELP[V-60] with *Xho*I and *EcoR*I restriction enzymes (*Xho*I-ELP[V-60]-ELP[V-60]-*EcoR*I), and ligated with mVenus (mV) and mTurquoise2 (mT) genes at the 5’- and 3’-ends of the ELP[V-60]-ELP[V-60] gene, respectively. The restriction enzymes for the mV gene were *BamH*I and *Xho*I (*BamH*I-mV-*Xho*I), while those for the mT gene were *EcoR*I and *Hind*III (*EcoR*I-mT-*Hind*III). We then inserted the ELP-TEMP gene into the pRSET_B_ vector (Invitrogen) for bacterial expression or the pcDNA3.1(-) vector (Invitrogen) for mammalian expression. To construct a reference indicator (ELP-REF), we replaced the ELP[V-60]-ELP[V-60] sequence in the ELP-TEMP gene with that of ELP[AV-60]-ELP[AV-60]. A plasmid containing a crowding indicator gene (crGE^27^) was a gift from Prof. Boersma. We transformed all the gene constructions into XL-10 Gold *E. coli* cells (200314, Agilent Technologies) and cultured in the Luria-Bertani (LB) broth medium with 100 µg/mL carbenicillin (Sigma-Aldrich) for 10–12 h at 37 °C, and then, we performed plasmid purification.

### Protein purification

We transformed *E. coli* strain JM109(DE3) (P9801, Promega) with the pRSET_B_ plasmids encoding ELP-TEMP or ELP-REF fused with an N-terminal polyhistidine tag by heat shock method at 42 °C for 45 s. We spread the transformants on an LB plate containing 100 µg/mL carbenicillin and incubated at 37 °C for overnight. We grew *E. coli* in a 200 mL LB medium containing 100 µg/mL carbenicillin at 23 °C for 3 days with gentle shaking at 120 rpm. We harvested the *E. coli* cells, suspended them in a phosphate buffered saline solution (PBS; T900, Takara Bio) containing a protease inhibitor cocktail (11873580001, Roche Diagnostics), and ultra-sonicated to lysate. We subsequently applied the proteins on the Ni–NTA agarose (30230, Qiagen) and eluted them with a TN buffer (10 mM Tris–HCl and 150 mM NaCl, pH 8.0) supplemented with 200 mM imidazole (099–00013, FUJIFILM Wako). Finally, we exchanged the solvent of the protein solution with a PBS solution (pH 7.4) by applying on a PD-10 desalting column (17085101, GE Healthcare) equilibrated with the same buffer. We concentrated the protein by ultrafiltration using a filter with a molecular weight cut-off of 50 kDa (UFC803024, Amicon Ultra-4, Merck Millipore), quickly froze the protein solution in liquid nitrogen, and stored them at − 80 °C.

### Fluorescence spectroscopy measurement

We performed fluorescence spectroscopy measurements by an FP-750 spectrofluorometer (JASCO) equipped with a temperature controller unit (ETC-272T, JASCO). We diluted proteins in a PBS solution (T900, Takara Bio) and loaded into a quartz cuvette. We took 5 min to wait for temperature equilibration at each temperature point before starting measurement. The excitation wavelength was 430 nm.

To investigate the effect of macromolecular crowding, we dissolved ELP-TEMP in a PBS solution containing Ficoll PM70 (F2878, Sigma-Aldrich). To examine the effect of salts, we added CaCl_2_, MgCl_2_, or 1 mM EDTA to a PBS solution, where 1 mM EDTA was used to achieve the condition of 0 mM CaCl_2_ or MgCl_2_. For NaCl and KCl, we added a salt to a 10 mM sodium phosphate buffer (pH 7.4). For pH dependence measurement, we prepared a mixture of 30 mM trisodium citrate and 30 mM borax adjusted to pH 8.0, 7.0, 6.0, and 5.0 by adding HCl.

To measure the fluorescence of ELP-TEMP in a cell suspension, we suspended ~ 5 million HeLa cells expressing ELP-TEMP in 0.5 mL of a PBS solution, and measured the fluorescence by a fluorescence spectrophotometer (F-7000, Hitachi-Hightech). For background correction, we measured the baseline from a suspension containing ~ 5 million untransfected HeLa cells. The excitation wavelength was 430 nm.

### Cell culture, transfection, and establishment of stable cell lines

We cultured HeLa cells in Dulbecco’s modified Eagle medium (DMEM; D6046, Sigma-Aldrich) supplemented with 10% fetal bovine serum (FBS; Biowest) at 37 °C in a 5% CO_2_ incubator. We seeded cells on in-house-made glass bottom dishes and grew up to 60–70% of cell confluence before performing transfection. For transfection, we mixed 2.0 µg of plasmids and 5.0 µg of polyethylenimine MAX (24765–1, Polysciences) in 200 µL of Opti-MEM medium (31985-070, Thermo Fisher Scientific), and incubated for 20 min at room temperature. Subsequently, we added the mixture to 1 mL of culture medium containing HeLa cells, and exchanged the culture medium at 6 h after the mixing. We cultured the cells for ~ 48 h and exchanged the medium to DMEM/F12 (11039-021, ThermoFisher Scientific) without phenol red before microscopy observation.

To establish a HeLa cell line stably expressing ELP-TEMP or ELP-REF, 2 days after transfection, we exchanged the culture medium with DMEM supplemented with 10% FBS and 500 µg/mL geneticin (G-418, 10131-035, Gibco). We kept culturing the cells until colonies formed. Finally, we isolated a fluorescent colony and grew up in the same medium to increase the cell number. After establishing the stable cell lines, we grew them with a DMEM medium containing 10% FBS and 200 µg/mL geneticin.

### Cell imaging

We performed cell imaging with an inverted microscope (Ti-2, Nikon) equipped with a confocal unit (Dragonfly 200, Andor Technology), a microscope objective (CFI Plan Apochromat λ 60 × Oil; numerical aperture, 1.40; Nikon), and a stage-top incubator with 5% CO_2_ supply (STXG-WSKMX, Tokai Hit). We used a 445 nm laser for excitation and collected the fluorescence emission through bandpass filters (ET480/40 nm and ET540/30 nm for mT and mV, respectively; Chroma). We captured the fluorescence images with an EMCCD camera (iXon Ultra, Andor Technology). The exposure time was 500 ms, and the binning size was 2 × 2 pixels. We measured fluorescence ratios of mV/mT cell by cell to calculate the average values and the standard deviation.

### Quick temperature rise with a local heat spot using carbon nanotubes (CNTs)

We prepared a suspension of 1 mg/mL multiwalled carbon nanotubes (CNTs) (average diameter, 170 nm; average length, 5–9 μm; 659258, Sigma-Aldrich) in a mixture of 0.5 mL of Tween20 and 1.5 mL of a buffer containing 20 mM HEPES, 25 mM KCl, and 5 mM MgCl_2_ (pH 7.0) as described in Ref.^35^. For imaging, we changed the culture medium of HeLa cells stably expressing ELP-TEMP to FluoroBrite DMEM (A1896701, ThermoFisher Scientific) supplemented with 10% FBS and GlutaMAX (35050-061, ThermoFisher Scientific). Subsequently, we dispersed 20 µL of the CNTs suspension into the culture medium. We used an inverted microscope (IX71, Olympus) equipped with a PlanApo 60X/1.40 oil-immersion objective lens (Olympus), a Light Engine (SPECTRA X, Lumencor), a stage-top incubator (INUB-ONICS, Tokai Hit), and a dual-view optics (W-View GEMINI, Hamamatsu photonics). We used a dichroic mirror of FF471/539-Di01 (Semrock) and an excitation filter of FF02-438/24 (Semrock). We used the dual-view optics equipped with a dichroic mirror (FF520-Di02, Semrock), emission filters of FF01-483/32 (Semrock) for mT and FF01-562/40 (Semrock) for mV, and a sCMOS camera (ORCA Flash4.0, Hamamatsu Photonics). The exposure time was 200 ms, and the binning of the sCMOS camera was 4 × 4 pixels. Before starting the experiment, we allowed CNTs to settle down for 30–60 min. To generate local heat, we irradiated a cluster of CNTs located near to several cells with a focused beam at 638 nm from a laser (Cube 635-20C, Coherent). For precisely synchronizing the laser, light engine, and sCMOS, we used a delay pulse generator (Sapphire Plus 9214 + ; Quantum Composers) coupled to them. The temperature was 34 °C, and the medium was in a 5% CO_2_ atmosphere.

### Estimation of the shift of transition temperature

To quantitatively evaluate the difference of transition temperatures in Fig. 3b, we compared a normalized fluorescence ratio given by

$$R_{{\text{N}}} (T) = \frac{R(T) - R(30)}{{R(40) - R(30)}},$$

where *R*(*T*) is a fluorescence ratio at temperature *T*, because the fluorescence ratio values were quite different between the nucleus and cytoplasm. To estimate the shift of transition temperature, we computed the interpolation of *R*_N_(*T*) by spline curves for the nucleus and cytoplasm, *R*_N,Int_(*T*, Nu) and *R*_N,Int_(*T*, Cyto), respectively, and we searched for an optimal temperature shift ∆*T* such that the loss function *L*

$$L = \sum\limits_{i} {\left[ {R_{{\text{N,Int}}} (T_{i} ,{\text{Nu}}) - R_{{\text{N,Int}}} (T_{i} - \Delta T,{\text{Cyto}})} \right]^{2} }$$

was minimal, where *T*_*i*_ is the *i*-th temperature point. Accordingly, the phase transition temperature in the cytoplasm was calculated to be 1.5 °C higher than that in the nucleus. The *p*-value of a null hypothesis that *R*_N_(*T*_*i*_) for the nucleus and cytoplasm showed the same mean values was 0.01. In contrast, the *p*-value of a null hypothesis that *R*_N,Int_(*T*_*i*_ − ∆*T*, Cyto) and *R*_N,Int_(*T*_*i*_, Nu) showed the same mean values was 0.51 as calculated by the PairedTTest module of Mathematica software (Wolfram Research).

### Heat production with chemically induced Ca^2+^ influx

We transiently co-transfected HeLa cells stably expressing ELP-TEMP with R-GECO, a genetically encoded Ca^2+^ indicator^37^ with the same transfection method mentioned above. We observed the cells with an inverted microscope (Ti-2, Nikon) equipped with a confocal unit (Dragonfly 200, Andor Technology), and the condition was the same in the cell imaging section mentioned above. We stimulated the cells with ionomycin (I-700, Alomone Labs) to the final concentration of 4 µM in DMEM/F12 (11039-021, ThermoFisher Scientific) by a home-made perfusion system. Medium temperature was 34 °C. For data analysis, we selected ROIs in the nucleus or cytoplasm of cells and used the nucleus or cytoplasm calibration curve in Fig. 3b for estimating temperature.

## Supplementary Information

Supplementary Information 1.

Supplementary Video 1.

Supplementary Video 2.

Supplementary Video 3.
