# Supplementary material for: A highly-sensitive genetically encoded temperature indicator exploiting a temperature-responsive elastin-like polypeptide
Source: Sci Rep. 2021 Aug 13;11:16519. doi: 10.1038/s41598-021-96049-5 (PMC8363741; doi:10.1038/s41598-021-96049-5)
Supplement: Supplementary file 1 — Supplementary Information 1. [file 41598_2021_96049_MOESM1_ESM.pdf]

SUPPLEMENTARY INFORMATION

A highly-sensitive genetically encoded temperature indicator exploiting a temperature-responsive elastin-like polypeptide

Cong Quang Vu[1,2], Shun-ichi Fukushima[2], Tetsuichi Wazawa[2], and Takeharu Nagai[1,2*]

[1] Graduate School of Frontier Biosciences, Osaka University, Suita, Osaka 565-0871, Japan
[2] SANKEN (The Institute of Scientific and Industrial Research), Osaka University, Ibaraki, Osaka 567-0047, Japan
[*]Correspondence: ng1@sanken.osaka-u.ac.jp

Supplementary movies

Supplementary movie 1：

Monitoring quick temperature rise in live HeLa cells with a local heat spot.

Supplementary movie 2:

Visualization of heat production from ionomycin-induced Ca^{2+} influx by ELP-TEMP.

Supplementary movie 3:

Monitoring the intracellular molecular crowding change with ionomycin stimulation.

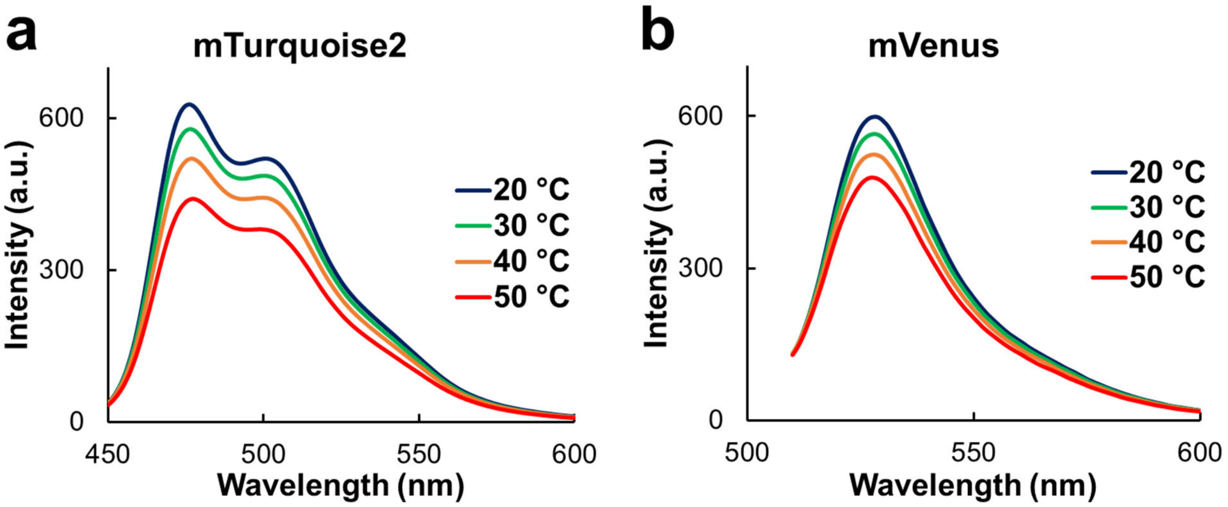

Figure S1. Temperature dependence fluorescence intensity of (a) mTurquoise2 and (b) mVenus. Purified proteins were dissolved in a PBS solution (pH 7.4) and measured their fluorescence spectrum at various temperatures. Excitation was 430 and 500 nm for mTurquoise2 and mVenus, respectively.

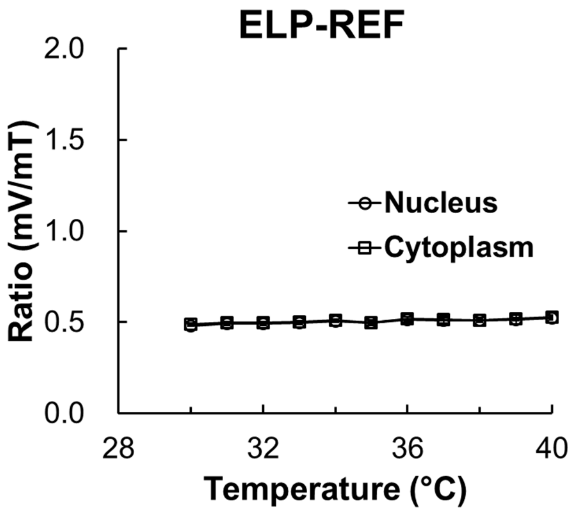

Figure S2. Confocal microscopy observation of live HeLa cells stably expressing ELP-REF. A plot of fluorescence ratio mV/mT of ELP-REF in the nucleus and cytoplasm against medium temperature. We captured fluorescence images with the same imaging condition as in Fig. 3 (see Methods for more detail). Data are mean ± SD (n = 16).

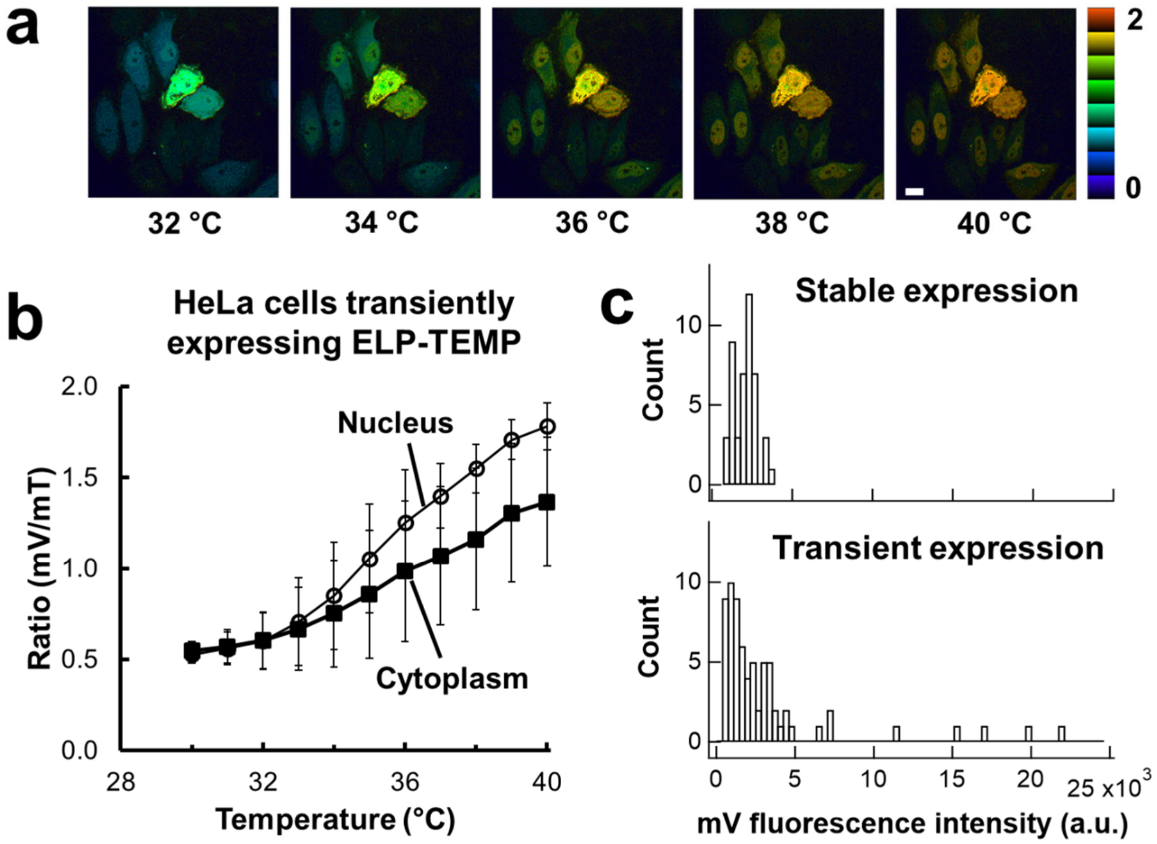

Figure S3. Confocal microscopy observation of live HeLa cells transiently expressing ELP-TEMP at various temperatures. (**a**) Pseudo-colored ratio images of HeLa cells transiently expressing ELP-TEMP at various temperatures. Fluorescence images were taken by the same imaging condition as in Fig. 3 (see Methods for more detail). The color bar indicates fluorescence ratio of mV/mT. Scale bar, 20 µm. (**b**) A plot of fluorescence ratio mV/mT of ELP-TEMP in the nucleus and cytoplasm against medium temperature. Data are mean ± SD (n = 11). (**c**) Histograms of mV fluorescence in ELP-TEMP by direct excitation mV at 514 nm between stable and transient expression of ELP-TEMP. The mV fluorescence intensity by direct excitation would present the concentration of ELP-TEMP in cells.

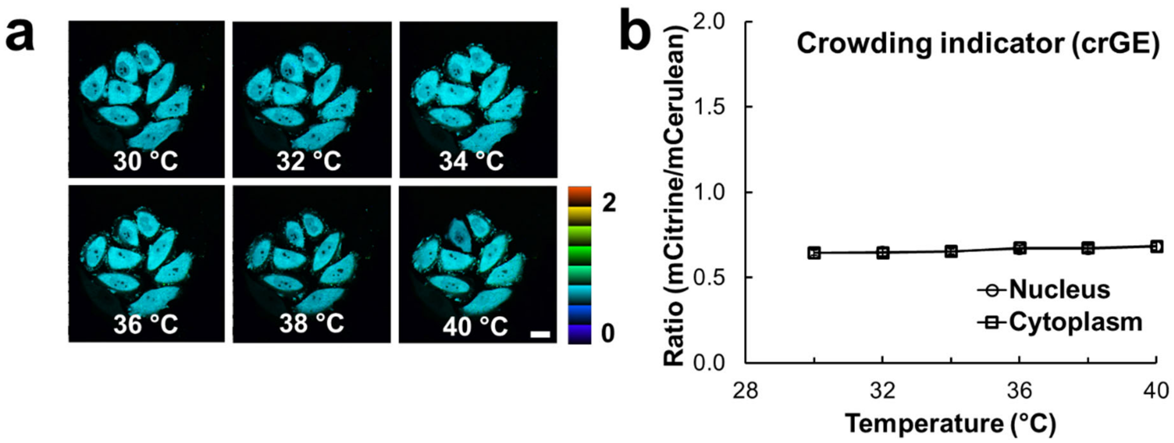

Figure S4. Confocal microscopy observation of live HeLa cells expressing a genetically encoded crowding indicator (crGE) at various medium temperatures. (**a**) Pseudo-colored ratio images of HeLa cells expressing crGE. Fluorescence images were taken by the same imaging condition as in Fig. 3 (see Methods for more detail). (**b**) A plot of fluorescence ratio mCitrine/mCerulean in the nucleus and cytoplasm against medium temperature. The color bar indicates fluorescence ratio of mCitrine/mCerulean. Scale bar, 20 µm. Data are mean ± SD (*n* = 8).

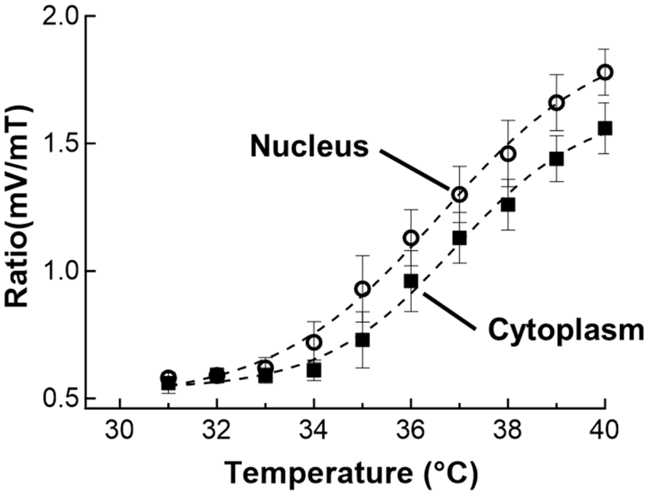

Figure S5. A plot of mV/mT ratio of ELP-TEMP in the nucleus and cytoplasm against temperature observed by the same microscope for the CNTs experiments. We observed HeLa cells stably expressing ELP-TEMP with a fluorescence microscope equipped with dual-view optics with a dichroic mirror (FF520-Di02, Semrock), emission filters of FF01-483/32 (Semrock) for mT and FF01-562/40 (Semrock) for mV, and a sCMOS camera (ORCA Flash4.0, Hamamatsu Photonics) (see Methods for more detail). Dash lines indicate sigmoidal fitting. Data are mean ± SD (n = 6).

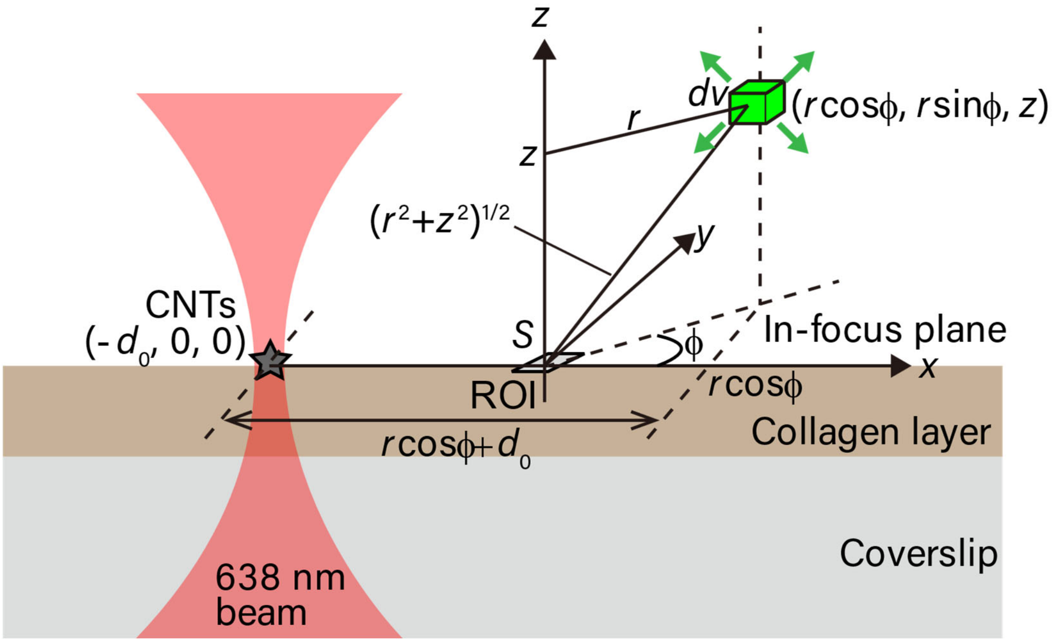

Figure S6. Schematic diagram for the estimation of uncertainty of the distance between a CNT cluster and a ROI. A CNT cluster at a position of ($-d_0$, 0, 0) (in Cartesian coordinates) was assumed to be irradiated with a focused 638-nm laser beam, and the fluorescence passing through the ROI was assumed to be detected. Because the measurement of temperature change in Fig. 4 was performed on a wide-field fluorescence microscope, the excitation light penetrated through the whole volume of a cell under observation. When the fluorescence intensity from a ROI was measured by a camera in the microscope, the intensity from a ROI should be contributed by fluorescence from fluorescent proteins off the ROI. Here we hope to estimate the fluorescence intensity emitted from an infinitesimally-small volume *dv* passing through the ROI with an area of *S* in the cylindrical coordinate. For simplicity, we supposed that the concentration of fluorescent protein was uniform in the cell, the whole cell was illuminated with the excitation light at a uniform power density, and the size of the ROI was negligibly small in comparison to the distance between the ROI and the volume *dv*. Let *F* be the total fluorescence intensity directing all direction emitted from a unit volume containing fluorescent protein, and we assumed that the in-focus plane was in the x-y plane. For the convenience of

integration, we considered the position of dv in cylindrical coordinates for the calculations below. By the inverse square law[1] the fluorescence intensity emitted from the volume dv passing through the area S, $F_{dv,S}dv$, is then described by

$$F_{dv,S}dv = \frac{F \cdot z \cdot S}{4\pi(r^2 + z^2)^{3/2}} rdrdzd\phi$$

Thus, the total fluorescence intensity passing through the area S, F_S, is calculated as

$$F_S = \int_V F_{dv,S} rdrdzd\phi ,$$

where V refers to the volume of a cell of our interest. To estimate the uncertainty of the distance d_0, we used the x-axis projection of the distance between the CNT cluster and the volume dv given by $d = |r\cos\phi - (-d_0)| = |r\cos\phi + d_0|$, and we weighted the error of distance $(d - d_0)$ by $F_{dv,S}\,dv$ to calculate its 2nd order moment $\langle(d - d_0)^2\rangle$ given by

$$\left\langle (d-d_0)^2 \right\rangle = \frac{\int_V (d-d_0)^2 F_{dv,S} rdrdzd\phi}{\int_V F_{dv,S} rdrdzd\phi} = \frac{\int_V (|r\cos\phi + d_0| - d_0)^2 F_{dv,S} rdrdzd\phi}{\int_V F_{dv,S} rdrdzd\phi} .$$

The thickness of HeLa cells was measured to be 16 ± 2 μm (n = 12) as observed by a confocal microscope. The longitudinal and transverse radius of the cell measured in Fig. 4 was measured to be 35 and 13 μm, respectively, and thus, we used the integral range of r from 0 to 24 μm, as the average. Because we used an objective lens with a numerical aperture of 1.40 in Fig. 4, we noted that the coverage of the incidence angle with the objective lens was near to 90° the aqueous medium. With the conditions as described thus far, we performed numerical integral calculations by using Mathematica software (Wolfram Research). Figure 4e shows plots for r = 5.6–31.2 μm. Based on the formulation above, the uncertainty of $\langle(d - d_0)^2\rangle^{1/2}$ was estimated to be 5.8, 7.0, 7.5 μm at r = 5.6, 15, and 31.2 mm, respectively.

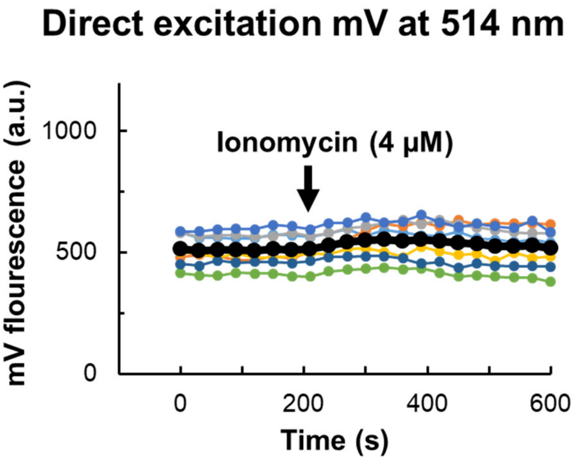

Figure S7. Investigation of the effect of ELP-TEMP concentration by direct excitation mV in ELP-TEMP under the stimulation of ionomycin. The mV fluorescence images were captured in the same experiment in Fig. 5c,d,e. The mV fluorescence intensity was taken from the same ROIs as in Fig. 5c,d,e. We used a 514 nm laser for direct excitation mV and collected the fluorescence emission through a bandpass filter (ET540/30nm; Chroma). We took the fluorescence images by an EMCCD camera (iXon Ultra, Andor Technology). Exposure time, 150 ms; binning size, 2×2 pixels. The arrow indicates the start of Ca^{2+} influx induced by ionomycin stimulation.

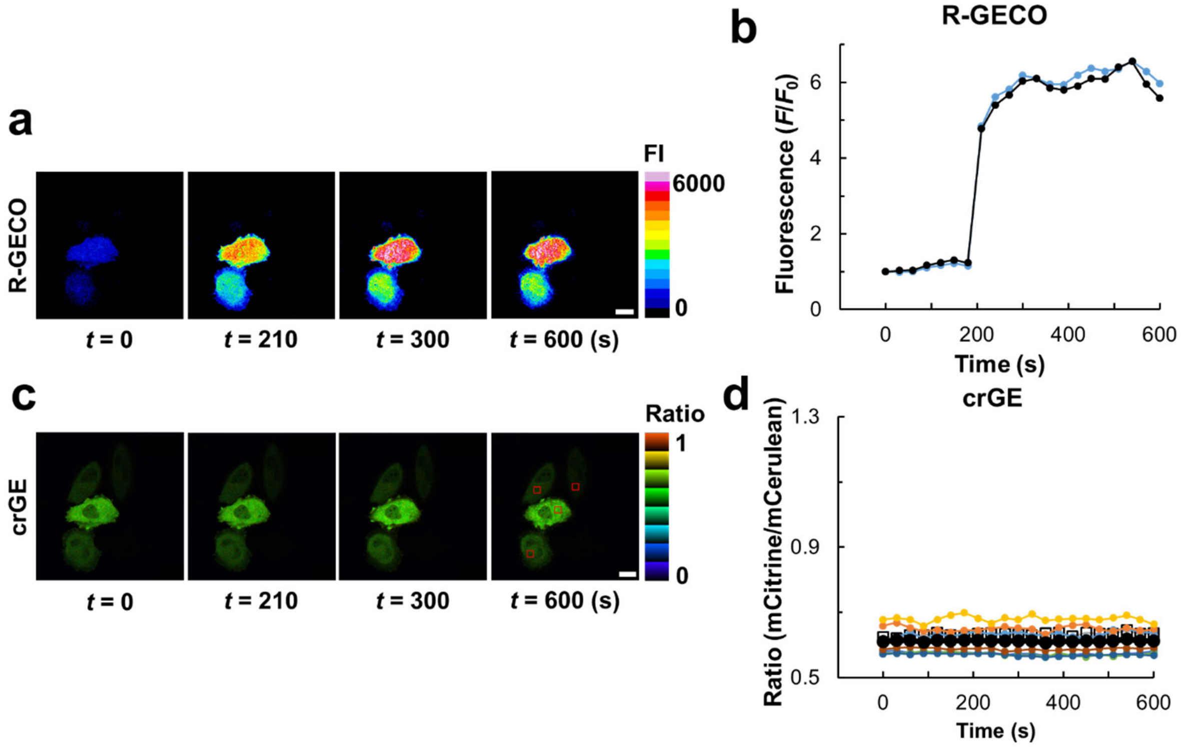

Figure S8. Investigation of the effect of ionomycin treatment on macromolecular crowding of HeLa cells transiently co-expressing crGE and R-GECO. (**a**) Pseudo-colored fluorescence images of R-GECO in responding to ionomycin stimulation. (**b**) A plot of fluorescence intensity (F/F_0) of R-GECO against time. (**c**) Pseudo-colored ratio images of crGE in responding to ionomycin stimulation. (**d**) A plot of fluorescence ratio of crGE against time. The observation was performed under the same confocal microscope in Fig 5. Ionomycin (4 µM) was added into the cell media by perfusion. Red squares indicate ROIs. The color bars indicate fluorescence intensity (FI) and ratio for (**a**) and (**c**), respectively. Medium temperature was 34 °C. Scale bars, 20 µm.

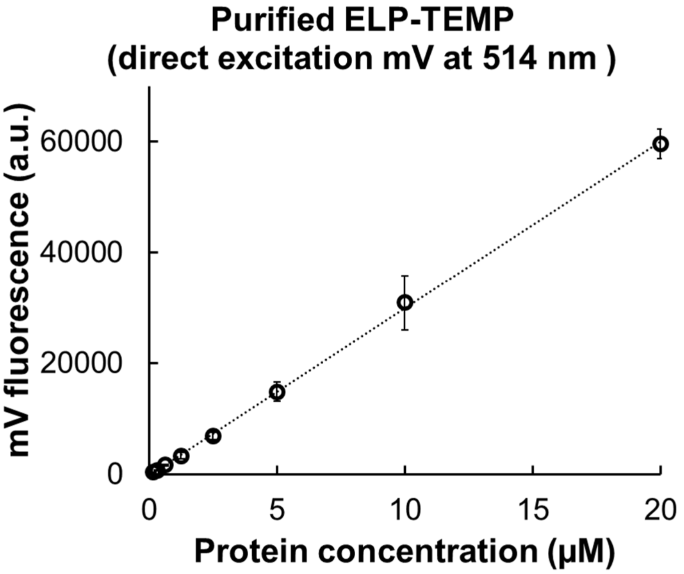

Figure S9. A plot of mV fluorescence intensity directly-excited at 514 nm in purified ELP-TEMP against the protein concentration observed by a confocal microscope. Purified ELP-TEMP was dissolved in a DMEM/F12 medium that used for cell imaging. The imaging condition was the same as that in Fig. 6a. In particular, we used a 514 nm laser for direct excitation mV and collected the fluorescence emission through a bandpass filter (ET540/30nm; Chroma). We captured the fluorescence images with an EMCCD camera (iXon Ultra, Andor Technology). The exposure time was 150 ms, and the binning size was 2×2 pixels. Data are mean ± SD (n = 3).

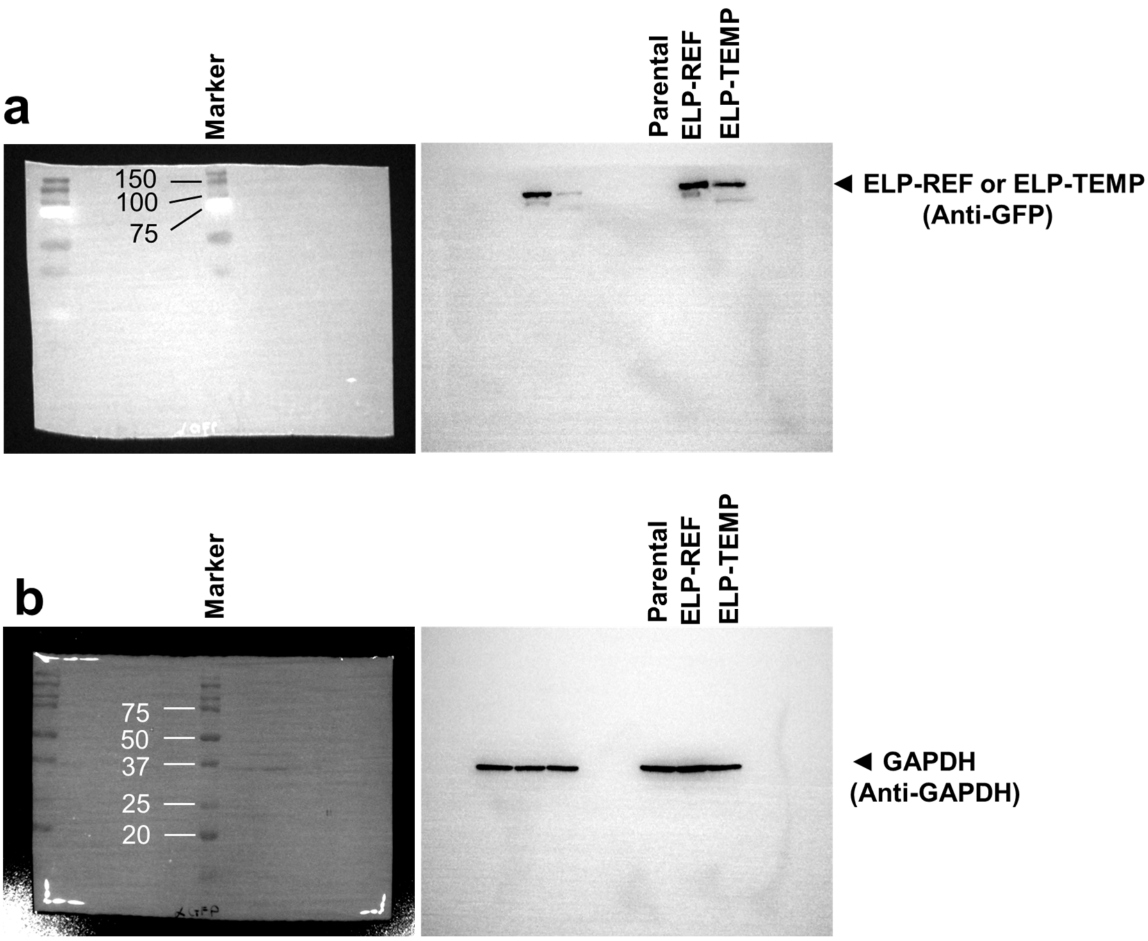

Figure S10. Western Blotting of stable HeLa cell lines expressing ELP-REF or ELP-TEMP detected with (a) anti-GFP and (b) anti-GAPDH. In this experiment, we aimed to examine the degradation of ELP-REF and ELP-TEMP in the stable HeLa cell lines. We performed SDS-PAGE of extracts from HeLa cells followed by Western blotting, and we took images of the membranes by a luminescence image analyzer (LAS-1000, Fujifilm). In panel (**a**), we detected the indicators with a primary anti-GFP (rabbit pAb, Cat #598, MBL, dilution 1:1000) and a secondary anti-rabbit IgG HPR conjugation (W401B, Promega, dilution 1:1000). In panel (**b**), as a loading control, we used a primary anti-GAPDH (mouse mAb, SC-32233, Santa Cruz, dilution 1:1000) and a secondary anti-mouse IgG HPR conjugation (W402B, Promega, dilution 1:1000). For the lanes of "Parental", "ELP-REF", and "ELP-TEMP", we took chemiluminescence images (right), in which we used a kit of ECL prime Western Blotting detection regents (RPN2232, GE Healthcare). For the marker (left) in panel (**a**), we took a red fluorescence image with a blue excitation light. The 75 kDa-band fluoresced, and other bands were dark because of light absorption. For the marker lane (left) in panel (**b**), we took a bright-field image with epi-illumination. "Parental" indicates extracts from HeLa cells without transfection. The numbers in the marker lanes indicate molecular weights (kDa) of the markers.

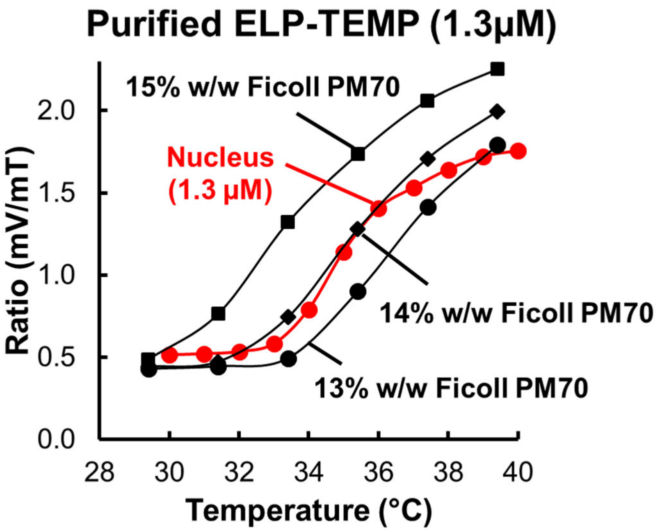

Figure S11. Optimization of the effect of Ficoll PM70 to the temperature response of ELP-TEMP. Purified ELP-TEMP was dissolved at a concentration of 1.3 μM in a PBS solution containing 13, 14, or 15% w/w Ficoll PM70. Interpolated data points for purified ELP-TEMP on the trajectories of 13, 14, and 15% w/w showed correlation coefficients of, respectively, 0.971, 0.993, and 0.952 with the fluorescence ratio of the nucleus (red points). The *p*-values of the null hypothesis that the fluorescence ratios of the nucleus and the interpolated data points from the purified ELP-TEMP showed the same mean values were 0.0035, 0.11, and 0.000097, for 13%, 14%, and 15% w/w, respectively, as calculated by the PairedTTest module of Mathematica software (Wolfram Research). For consistency with the microscopy data, we calculated the integral of $F(\lambda)T(\lambda)$ for mT and mV, where F is a fluorescence emission spectrum and T is a composite spectral transmittance of a bandpass filter and a dichroic mirror. The integration wavelengths were 457–500 nm and 526–552 nm for mT and mV, respectively. The temperature response of ELP-TEMP in the nucleus (red line) was obtained from microscopy data in Fig. 3b. The excitation was 430 nm.

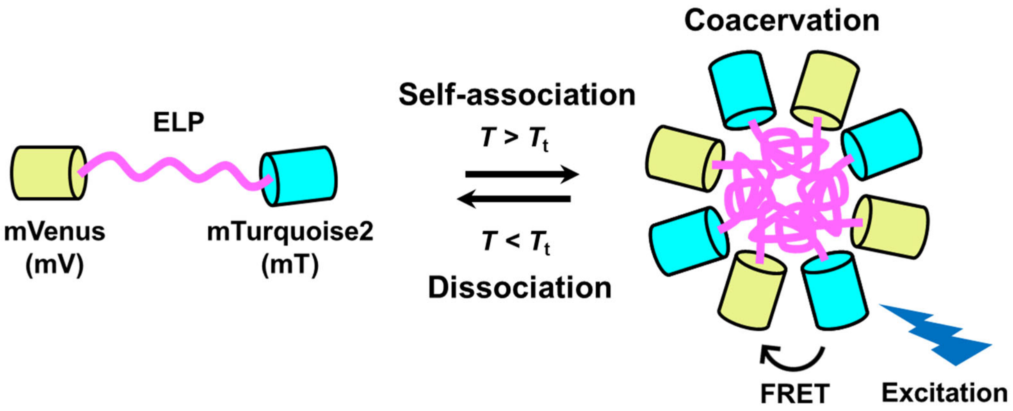

Figure S12. Schematic illustration of proposed temperature-sensing mechanism of ELP-FP fusion proteins exploiting lower critical solution temperature (LCST) of an ELP. ELP has coacervation property that is self-assembling of molecules with ordered structures dependent on increasing of temperature. At temperatures below T_t, the conformation of ELP-FP fusion proteins would be largely extended and dispersed in the solution so that FRET from mT to mV occurs at a low efficiency. At temperatures above T_t, the ELP moiety in the ELP-FP fusion proteins would undergo the conformation change and self-assembly into compacted coacervate so that the average distance between mT and mV becomes small, and FRET between them occurs at a high efficiency. In addition, because mT and mV are hydrophilic and ELP is hydrophobic, mT and mV may be largely exposed to the solvent and the ELP coacervate may be largely covered by mT and mV.

Table S1. Relative temperature sensitivity (S_T) of some non-genetically encoded fluorescent nanothermometers for intracellular thermometry

Nanothermometers	Materials	Measurement method	S_T (%/°C)	Ref.
ER thermo yellow	Fluorescent dye	Fluorescence intensity	3.9	[2]
Mito thermo yellow	Fluorescent dye	Fluorescence intensity	2.0–2.8	[3]
Mito-RTP	Fluorescent dye	Fluorescence intensity ratio	2.7	[4]
Mito-TEMP 2.0	Fluorescent dye	Fluorescence intensity ratio	5.4	[5]
Eu-TTA within micropipette	Fluorescent dye	Fluorescence intensity	2.7	[6]
Thermosensitive nanovesicles	Fluorescent dye and lipids	Fluorescence intensity	10–22	[7]
Walking nanothermometer	Fluorescent dyes and polymers	Fluorescence intensity ratio	2.2	[8]
Ratiometric nanothermometer	Fluorescent dyes and polymers	Fluorescence intensity ratio	2.2	[9]
Fluorescent thermometer nanosheets	Fluorescent dyes and polymers	Fluorescence intensity ratio	3.4	[10]
Ultra-long-lived luminescent nanocapsule	Fluorescent dyes and polymers	Fluorescence life time	7.5	[11]
Lanthanide-Bearing Polymeric Micelles	Fluorescent dyes and polymers	Fluorescence intensity ratio	1.7	[12]
Quantum dot (QD655)	Quantum dot	Fluorescence intensity ratio	6.2	[13]
NaYF4 nanothermometer	Infrared luminescent nanoparticles	Fluorescence intensity ratio	1.9	[14]

References

1 Warren, J. S. *Modern Optical Engineering: The Design of Optical Systems, Fourth Edition*. 4th ed. edn, (McGraw-Hill Education, 2008).

2 Arai, S., Lee, S.-C., Zhai, D., Suzuki, M. & Chang, Y. T. A Molecular Fluorescent Probe for Targeted Visualization of Temperature at the Endoplasmic Reticulum. *Sci. Rep.* **4**, 6701, doi:10.1038/srep06701 (2014).

3 Arai, S. *et al.* Mitochondria-targeted fluorescent thermometer monitors intracellular temperature gradient. *Chem. Commun. (Camb.)* **51**, 8044-8047, doi:10.1039/c5cc01088h (2015).

4 Homma, M., Takei, Y., Murata, A., Inoue, T. & Takeoka, S. A ratiometric fluorescent molecular probe for visualization of mitochondrial temperature in living cells. *Chem. Commun.* **51**, 6194-6197, doi:10.1039/C4CC10349A (2015).

5 Huang, Z., Li, N., Zhang, X. & Xiao, Y. Mitochondria-Anchored Molecular Thermometer Quantitatively Monitoring Cellular Inflammations. *Anal. Chem.* **93**, 5081–5088, doi:10.1021/acs.analchem.0c04547 (2021).

6 Suzuki, M., Tseeb, V., Oyama, K. & Ishiwata, S. i. Microscopic detection of thermogenesis in a single HeLa cell. *Biophys. J.* **92**, L46-L48, doi:10.1529/biophysj.106.098673 (2007).

7 Sou, K., Chan, L. Y., Arai, S. & Lee, C.-L. K. Highly cooperative fluorescence switching of self-assembled squaraine dye at tunable threshold temperatures using thermosensitive nanovesicles for optical sensing and imaging. *Sci. Rep.* **9**, 17991, doi:10.1038/s41598-019-54418-1 (2019).

8 Oyama, K. *et al.* Walking nanothermometers: spatiotemporal temperature measurement of transported acidic organelles in single living cells. *Lab on a Chip* **12**, 1591-1593, doi:10.1039/C2LC00014H (2012).

9 Takei, Y. *et al.* A Nanoparticle-Based Ratiometric and Self-Calibrated Fluorescent Thermometer for Single Living Cells. *ACS Nano* **8**, 198-206, doi:10.1021/nn405456e (2014).

10 Oyama, K. *et al.* Single-cell temperature mapping with fluorescent thermometer nanosheets. *J. Gen. Physiol.* **152**, doi:10.1085/jgp.201912469 (2020).

11 Su, X. *et al.* Lifetime-based nanothermometry in vivo with ultra-long-lived luminescence. *Chem. Commun.*, doi:10.1039/D0CC04459H (2020).

12 Piñol, R. *et al.* Real-time intracellular temperature imaging using lanthanide-bearing polymeric micelles. *Nano Lett.*, doi:10.1021/acs.nanolett.0c02163 (2020).

13 Tanimoto, R. *et al.* Detection of Temperature Difference in Neuronal Cells. *Sci. Rep.* **6**, 10, doi:10.1038/srep22071 (2016).

14 Sekiyama, S. *et al.* Temperature Sensing of Deep Abdominal Region in Mice by Using Over-1000 nm Near-Infrared Luminescence of Rare-Earth-Doped NaYF4 Nanothermometer. *Sci. Rep.* **8**, 16979, doi:10.1038/s41598-018-35354-y (2018).
